# Fine mapping chromatin contacts in capture Hi-C data

**DOI:** 10.1186/s12864-018-5314-5

**Published:** 2019-01-23

**Authors:** Christiaan Q Eijsbouts, Oliver S Burren, Paul J Newcombe, Chris Wallace

**Affiliations:** 1 Department of Medicine, University of Cambridge, Addenbrooke’s Hospital, Hills Road, Cambridge, UK; 2 MRC Biostatistics Unit, Institute of Public Health, University Forvie Site, Robinson Way, Cambridge, UK; 3 Current address: Wellcome Centre for Human Genetics, Nuffield Department of Medicine, University of Oxford, Roosevelt Drive, Oxford, UK

**Keywords:** Capture Hi-C, Chromatin conformation, Bayesian statistics, Variable selection

## Abstract

**Background:**

Hi-C and capture Hi-C (CHi-C) are used to map physical contacts between chromatin regions in cell nuclei using high-throughput sequencing. Analysis typically proceeds considering the evidence for contacts between each possible pair of fragments independent from other pairs. This can produce long runs of fragments which appear to all make contact with the same baited fragment of interest.

**Results:**

We hypothesised that these long runs could result from a smaller subset of direct contacts and propose a new method, based on a Bayesian sparse variable selection approach, which attempts to fine map these direct contacts. Our model is conceptually novel, exploiting the spatial pattern of counts in CHi-C data. Although we use only the CHi-C count data in fitting the model, we show that the fragments prioritised display biological properties that would be expected of true contacts: for bait fragments corresponding to gene promoters, we identify contact fragments with active chromatin and contacts that correspond to edges found in previously defined enhancer-target networks; conversely, for intergenic bait fragments, we identify contact fragments corresponding to promoters for genes expressed in that cell type. We show that long runs of apparently co-contacting fragments can typically be explained using a subset of direct contacts consisting of <10*%* of the number in the full run, suggesting that greater resolution can be extracted from existing datasets.

**Conclusions:**

Our results appear largely complementary to those from a per-fragment analytical approach, suggesting that they provide an additional level of interpretation that may be used to increase resolution for mapping direct contacts in CHi-C experiments.

**Electronic supplementary material:**

The online version of this article (10.1186/s12864-018-5314-5) contains supplementary material, which is available to authorized users.

## Background

The three-dimensional structure of the genome influences gene expression at varying levels of scale [1]. Multi-megabase compartments of active and inactive chromatin, as well as topologically-associated domains (TADs) spanning hundreds of kilobases, can be readily identified by mapping physical interactions using genome-wide chromatin conformation capture techniques (Hi-C) [2, 3]. However, as Hi-C quantifies interactions between all possible pairs of regions in the genome (e.g. *Hind*III fragments) via massively parallel sequencing, it is inefficient at characterizing individual enhancer-promoter interactions in great depth. To explore such regulatory interactions in detail, the more recently developed Capture Hi-C (CHi-C) method targets sequencing efforts toward interactions between pre-defined regions of interest (“baits”, e.g. *Hind*III fragments overlapping gene promoters) on one end, and all other regions (“prey”) on the other [4, 5].

CHi-C has enabled identification of contacts made by promoters in primary human cells [5, 6]. The contact maps thus generated show a tendency for multiple contiguous fragments to be linked with the same promoter [7, 8], but it is not clear whether enhancers overlapping all these fragments or only a subset of them are directly relevant to the promoter’s regulation. Conversely, the same enhancer region can appear to interact with promoters of multiple genes [9, 10], while it remains unclear whether this reflects coregulation of these genes [11]. Either phenomenon could also be caused by a lack of resolution in these maps, which are typically constrained by the restriction enzyme used (e.g. *Hind*III produces fragments of median length 4kb). Given that typical enhancers and promoters are considerably shorter than a single *Hind*III fragment [12], we hypothesised that collateral contacts may be identified along with the direct enhancer-promoter contacts they neighbour. Such collateral contacts might result from a bait traversing the regions around its primary target via Brownian motion, potentially during the formation of loops [13] or from inaccuracies in the cross-linking of proximal regions during the CHi-C procedure [14, 15].

**Fig. 1:**
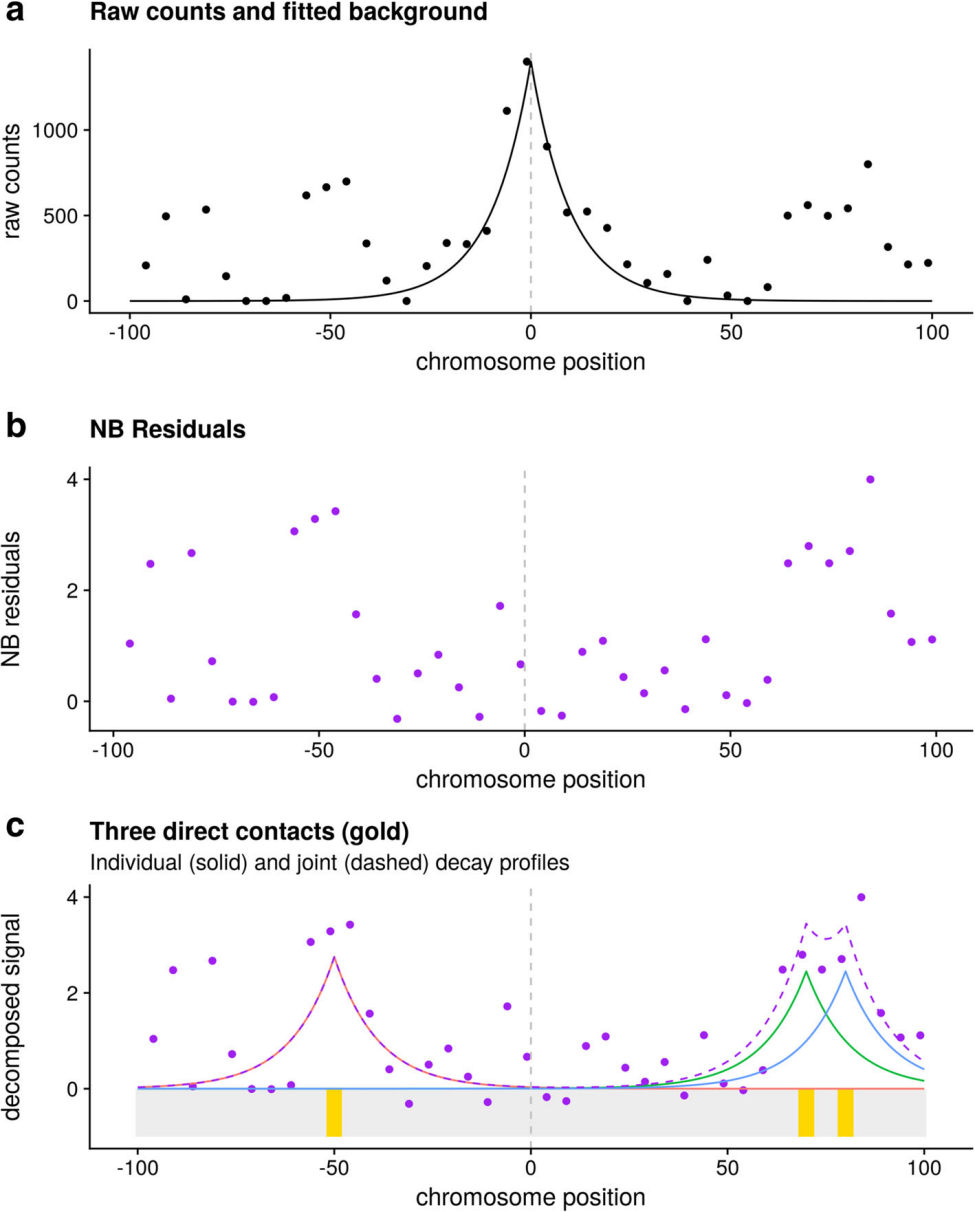
Schematic example of analysis in a single CHi-C region. **a** raw counts derived from the CHi-C experiment and the decay profile around the bait estimated by negative binomial (NB) regression. **b** residuals after NB regression represent the signal after adjusting for expected exponential decay around the bait. These residuals can be compared against a null distribution to generate *p*-values, in the same way CHiCAGO scores are generated – separately for each fragment, independent of local patterns in the signal. **c** Peaky extends the inference by considering the joint distributions of residuals across the region. This example shows three proposed direct contacts which could be jointly responsible for the spatial distribution of NB residuals, with solid lines indicating the individual decay functions fit in our joint model and the dashed line their predicted joint effect. The position of the bait fragment is indicated by the dotted line and chromosome position is shown in kb relative to the bait. Peaky inference at each prey fragment is based on the marginal posterior probability of a contact (MPPC), defined as the proportion of MCMC samples in which that fragment was selected as a direct contact with a positive peak height

The CHi-C signal around any given bait is represented by counts of read pairs (“counts”) linking that bait fragment to each of its neighbouring prey fragments. The signal exhibits a characteristic exponential decay around the location of the bait (Fig. 1a), thought to reflect Brownian motion rather than biologically interesting interactions. Existing approaches for calling interactions from CHi-C data first fit a regression model to these counts. The model estimates the expected rate of decay by distance from the baited fragment, while accounting for other bait- or prey-specific factors, such as capture efficiency and enrichment bias. Then, the count for each individual bait-prey fragment pair is considered, and those whose count is substantially above that predicted by the regression model are identified (Fig. 1b) [6, 9]. We noted that CHi-C signals often appeared spatially auto-correlated around interacting prey, in their raw as well as their regression-adjusted form. We sought to use this information to improve resolution of CHi-C contact maps. We hypothesised that joint modelling of neighbouring prey fragments would allow direct contacts to be distinguished from collateral contacts under the assumption that the CHi-C signal peaks at directly contacted preys and gradually decays amongst neighbouring fragments (Fig. 1c).

Here, we propose a statistical model in which, for any given bait, the expected CHi-C signal at each prey is expressed as a sum of contributions from a sparse set of fragments directly contacting that bait. This decomposition model allows us to view the CHi-C signal at each prey in the context of the signals in its local environment. We fit the model through reversible jump Markov Chain Monte Carlo (RJMCMC) to identify primary contacts in published CHi-C data from two cell types, non-activated and activated CD4 ^+^ T cells [8].

## Results

### A spatial model for CHi-C data

In common with other approaches [6, 9], we first process the read count data from CHi-C using a negative binomial (NB) model to adjust for fragment-specific effects as well as the decay in counts around the bait (Fig. 1a, see “Methods” section). We then define a joint model for the resulting standardised residuals, which we call “NB residuals” (Fig. 1b). We assume that these NB residuals follow a normal distribution with unit variance (owing to their standardisation) and, in the absence of interactions, zero mean. Where either direct or collateral contacts exist, we propose the NB residuals still follow a normal distribution with unit variance, but with a non-zero mean which we expect is positive. These assumptions are compatible with observed data (see below). Focusing on a single bait, *b*, and its nearest *F*_*b*_ neighbouring prey fragments, let *Y*_*bp*_ be the observed NB residual for prey fragment *p*. Then

1 $$  Y_{bp} \sim N(\mu_{bp}, 1) \text{ for} \,p=1,\ldots,F_{b}  $$

where *μ*_*bp*_ is the expected NB residual. We assume that a direct contact between *b* and a fragment *p* causes *μ*_*bp*_ to increase by some value *β*_*bp*_, the magnitude of which reflects the strength of interaction. In the absence of any other contacts we would simply have *μ*_*bp*_=*β*_*bp*_. However, we also assume that a direct contact at another fragment, *q*, can affect *μ*_*bp*_, particularly when it is near to *p*. Such additional contacts increase *μ*_*bp*_ by

2 $$  \delta(p,q;\beta_{bq}, \omega) = \beta_{bq} \times \exp(-\omega\ d(p,q))  $$

where *β*_*bq*_ captures the strength of the interaction at *q* and *d*(*p*,*q*) is the absolute linear distance between the midpoints of fragments *p* and *q*, with parameter *ω* (assumed fixed and known) controlling the rate of decay.

The exponential form in (2) was chosen by examining model fits with this and other possible forms of decay functions to a subset of baits. The value of *ω* was fixed at 10^−4.7^, chosen from a range of values tried because it produced the best fit to our data (full details in Additional file 1).

Thus, *μ*_*bp*_ can be expressed as a sum of contributions:

3 $$  \mu_{bp}= \sum_{q: \gamma_{bq}=1} \beta_{bq} \times \exp(-\omega\ d(p,q))  $$

where the sum is taken over all fragments *q* in some neighbourhood of the bait *b*, and *γ*_*bq*_ is a latent indicator variable, taking the value 1 if there is a direct contact between fragments *b* and *q* (i.e. *β*_*bq*_≠0) and 0 otherwise.

We provide functions to implement this model in the R package Peaky, available from http://github.com/cqgd/pky.

### Inference of bait-prey interactions

We fit the model using an RJMCMC sampler, R2BGLiMS [16]. For each bait *b*, the distribution of sampled coefficients $\phantom {\dot {i}\!}\beta _{b1},\ldots,\beta _{{bF}_{b}}$ reflect the posterior distribution of contact strengths between the bait *b* and its neighbouring preys. Given our prior assumption that contacts lead to increased rather than decreased counts, we decided not to use the common marginal posterior probability of inclusion, defined as the proprtion of samples in which *β*_*bp*_≠0. Instead, we defined an analogous statistic: the marginal posterior probability of a contact (MPPC) between bait *b* and prey *p*, as the proportion of sampled models in which *β*_*bp*_>0, and use this as the primary statistic for inference.

### Reproducibility of MPPC calls on replicate data from macrophages

**Fig. 2:**
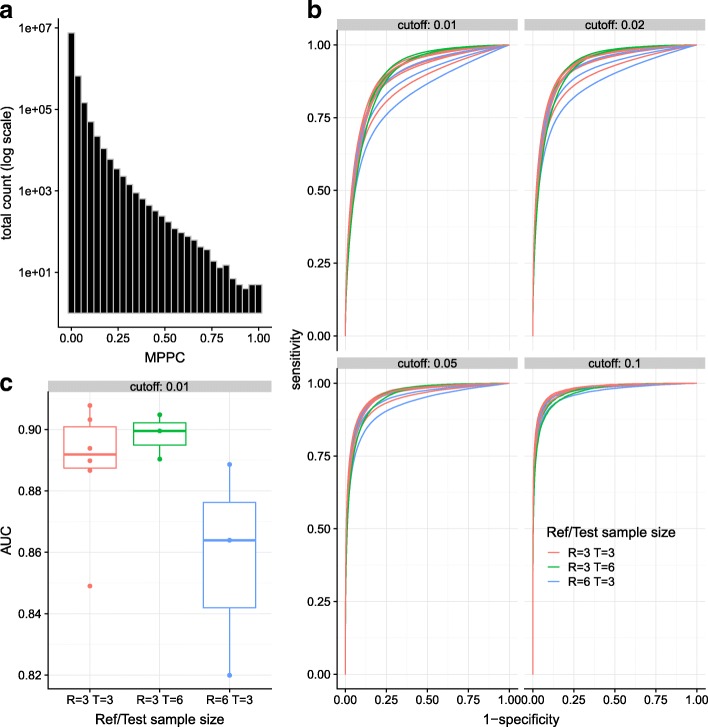
Reproducibility analysis of MPPC in macrophage dataset. **a** distribution of MPPC in all reference and test datasets. **b** ROC curves from calls in test data according to “true negatives” in reference data at MPPC ≤0.01 and “true positives” at MPPC above the cutoff shown for different reference and test data sample sizes. **c** distribution of AUC values calculated from each ROC curve

Previous analysis has shown strong similarities between the interactomes of different macrophages [8]. In order to examine the reproducibility of MPPC-based calls, we used macrophage data which had been collected on three stages of differentiation from three samples, giving results from nine individual samples [8]. As our intention was to apply our method to CD4 ^+^ T cell data from three samples, we combined data to compare calls from non-overlapping sets of three or six samples, each derived from one or two individuals across all developmental stages. We constructed all 12 possible ordered pairs from these data that did not contain the same individual twice, labelling one member “reference” and the other “test”. We ran peaky independently on each dataset, and stored the MPPC for each bait-prey pair on chromosomes 1–10. The distribution of MPPC was similar in each dataset, with an overall 83% of values <0.01 and 99% <0.1 (Fig. 2a). We called “true negatives” in each reference dataset at MPPC ≤0.01 and “true positives” at MPPC >*α* for *α*=0.01,0.02,0.05,0.1, then drew ROC curves to assess reproducibility in each corresponding test dataset (Fig. 2b). Reproducibility was best when both test and reference datasets were the same size, and worst when the reference data was bigger than the test data, as might be expected when attempting to detect contacts in a less powerful dataset. Overall, AUC values were very good, ranging from 0.80–0.92 (Fig. 2c) with higher values seen when higher alpha threshold were used to define “true positives”.

### Application to CHi-C data from activated and non-activated CD4 ^+^ T cells

We applied the above model to four parallel data sets generated from CD4 ^+^ T cells: two from non-activated cells (“non”) cultured for four hours in buffer and two from activated cells (“act”) cultured for four hours with anti-CD3/CD28 beads, all previously analysed with CHiCAGO [8]. We chose to use two cell types so we could check any results were representative rather than specific to a single dataset (which might indicate over-fitting), and chose these cell types specifically because of availability of external datasets for biological validation. Each pair consisted of a *promoter capture* set, with 22,032 bait fragments representing the promoters of 28,007 unique annotated genes (16,116 baits representing 17,731 protein coding genes), and a *validation capture* set, with 945 bait fragments that were preys contacting baits in the promoter dataset according to analysis using the standard CHiCAGO pipeline [6] in CD4 ^+^ T cells, megakaryocytes or erythroblasts [8]. Note that the validation set are used here to provide a complementary dataset whose true contacts would be expected to have alternative biological characteristics than the promoter capture set, owing to the opposite fragment being captured. The validation capture array was designed before peaky was conceived, on the basis of CHiCAGO scores only. Thus, because baits were not selected into the validation data on the basis of MPPC they cannot be used to validate the MPPC itself. We pre-processed raw counts from each dataset separately to generate NB residuals. QQ plots showed that our assumptions of central normality and a long right tail were met (Additional file 2: Figure S1).

Suggested practice by the authors of CHiCAGO is to declare “significant” interactions when CHiCAGO scores exceed 5 [6]. We followed this advice, and focused on baits which had at least one prey fragment with a CHiCAGO score >5 within a window of at least 10 mb around the bait (5 mb either side). We fitted the joint model described by (1) to the NB residuals within these windows for each bait separately in two parallel RJMCMC chains, running additional iterations until the correlation between MPPC from each chain exceeded 0.75. This was achieved for over 94% of baits within 20 million iterations (Table 1, Additional file 2: Figure S2), and we focus on our inference of these below. The union of samples from both chains was used for inference.

**Table 1:** Number and % of baits for which correlation between MPPC between two parallel runs exceeded 0.75

Experiment	Total baits	n. *ρ*>0.75	% *ρ*>0.75
Promoter, non	13078	12528	95.8
Promoter, act	13319	12785	96.0
Validation, non	648	622	96.0
Validation, act	706	688	97.5

The total baits is the number for which at least one prey fragment has a CHiCAGO score > 5

### MPPC provides additional information for distinguishing biologically plausible contacts

We first compared the MPPC and the CHiCAGO scores for each bait-prey pair. We noted that the CHiCAGO score decayed more rapidly with increasing distance from the bait fragment compared to the MPPC (Additional file 2: Figure S3), presumably reflecting, in part, the different approaches taken to long-distance contacts. CHiCAGO deliberately down-weights the significance of longer distance (with weights learned based on reproducibility of signals across technical replicates). As our intention is to fine-map longer runs of contacts identified by CHiCAGO, we chose not to apply any down-weighting in order to avoid doubly penalising them. We also noted that the MPPC and CHiCAGO score were positively correlated (Spearman’s *ρ*>0.23; Additional file 2: Figure S4), although a substantial fraction of bait-prey pairs showed high CHiCAGO scores and low MPPC or *vice versa*. We therefore investigated whether one measure alone, or both together, were better at predicting biologically plausible contacts using a variety of measures. We considered that direct contacts from baits in the promoter capture set should be more likely among prey that overlap active chromatin states or that corresponded to published CD4 ^+^ T cell promoter-enhancer networks [17], and in the validation set among prey that contain a gene promoter or the promoter of a more strongly expressed gene. We found that for all of these measures, regression models indicated that either MPPC and CHiCAGO scores together (7 cases) or MPPC alone (1 case) were best able to predict these features (Table 2). In all cases, measures of biological plausibility increased with increasing MPPC at any CHiCAGO threshold, suggesting that MPPC can be used to discriminate between fragments with similar CHiCAGO scores (Additional file 2: Figure S5). This suggested that MPPC and CHiCAGO could be used together to better predict biologically plausible direct contacts than a model which considers each bait-prey pair independently, such as CHiCAGO.

**Table 2:** *Δ*BIC from the intercept-only model for four measures of biological plausibility of contacts

Model	Non-activated	Activated
**a: Promoter: match to Cao et al.**		
MPPC	−338.0	−245.8
CHiCAGO	−332.2	−331.4
MPPC + CHiCAGO	^*a*^−411.6	^*a*^−358.8
**b: Promoter: link to active chromatin**		
MPPC	−1134.2	−812.3
CHiCAGO	−659.5	−560.1
MPPC + CHiCAGO	^*a*^−1231.1	^*a*^−924.0
**c: Validation: overlap baited promoter**		
MPPC	−404.8	−347.9
CHiCAGO	−430.0	−419.2
MPPC + CHiCAGO	^*a*^−541.7	^*a*^−499.0
**d: Validation: expression at linked promoter**		
MPPC	−1571.0	^*a*^−1329.2
CHiCAGO	−871.9	−430.2
MPPC + CHiCAGO	^*a*^−1640.3	−1318.7

The best fitting model (lowest *Δ*BIC) is highlighted by ^*a*^. **a**–**d** are defined in full in the “Methods” section. Briefly,

**a** whether the bait-prey pair corresponds to published CD4 ^+^ T cell promoter-enhancer networks [17];

**b** whether the prey fragment overlaps active chromatin states defined by [7];

**c** whether the prey overlaps a gene promoter;

**d** the level of expression of a gene associated with the prey fragment. In all cases, a robust clustered model was used to account for repeated observations at the prey fragment

### MPPC can be used to prioritise direct contacts amongst long runs

**Fig. 3:**
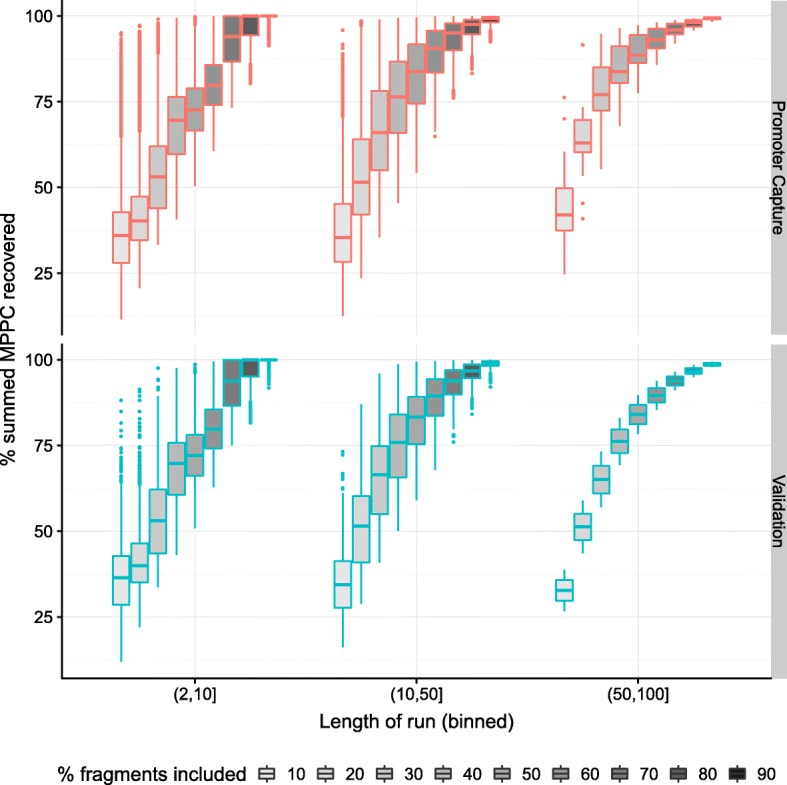
Runs of fragments with CHiCAGO scores >5 were binned by length (x-axis), and fragments within runs ordered by decreasing MPPC. Boxplots show the proportion of total posterior support captured by including increasingly larger subsets of the ordered fragments. For longer runs (>10 fragments), the majority (>50*%*) of the posterior, as quantified by the summed MPPC across the run, is generally be captured by a minority (<10*%*), of the fragments

The median number of prey fragments per bait identified by a CHiCAGO score >5 ranged from 7–8, but with a maximum over 200 (Additional file 2: Figure S6, Table S1). In comparison, our model tended to have a slightly greater expected number of contacts per bait when the CHiCAGO count was low (many of these related to fragments with CHiCAGO scores ∈ [ 3,5)), but many fewer when the CHiCAGO count was high (Additional file 2: Figure S6). To enable discussion of longer runs of fragments with high CHiCAGO scores, we define a “stretch” of length *n* to be a series of *n* adjacent fragments with CHiCAGO scores >5. In the longest stretches of length 50 or more, our model estimated the expected number of direct contacts to be ∼7% of the number of prey (Additional file 2: Figure S7). The posterior was spread over a larger number of fragments than the number of expected contacts, but it was not uniform, and we found that the majority of the posterior was often concentrated within a minority of the fragments: for example, within stretches of length 50 or more, a median of 76% of the corresponding sum of posterior probabilities over all fragments could be captured by just the top 30% of the fragments ranked by MPPC, while 90% of the posterior could be captured by the top 50% of the fragments (Fig. 3).

This suggested that long stretches could result from direct contacts at a small subset, and that our joint model could distinguish these, ranking some as more probable direct contacts than others. As the true sets of direct contacts are unknown, we again used external data to assess whether this prioritisation corresponded to fragments with more biologically supportable characteristics. We found that, within these stretches, the MPPC remained significant predictors of whether fragments corresponded to biologically plausible features across all run lengths (Additional file 2: Table S2).

**Fig. 4:**
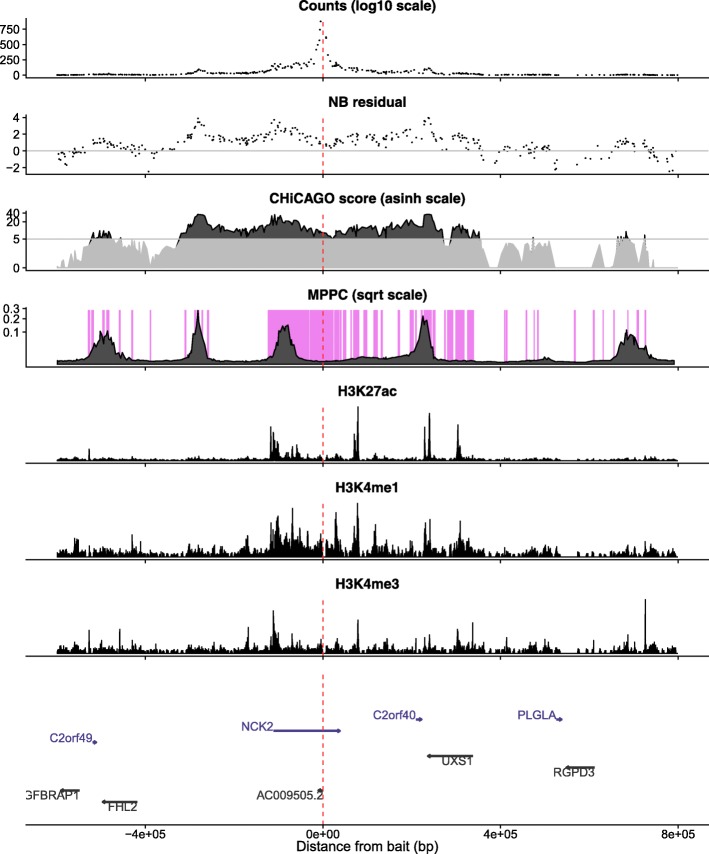
Illustrative example of analysis in the region of the a bait for the antisense gene AC009505.2 and an alternative promoter of *NCK2* in non-activated CD4 ^+^ T cells. The top three panels show raw counts, adjusted residuals, CHiCAGO scores, and MPPC. The MPPC is overlaid on shading highlighting regions of active chromatin derived by aggregating states from CHROMHMM analysis ChIP-seq data from the same cell type, as previously published [7]. Note that the long run of fragments to the left of the promoter with high CHiCAGO scores have MPPC profiles which visually cluster into two groups, each corresponding to regions with active chromatin marks (i.e. they overlie shaded regions). The next three panels show three examples of this CHiP-seq data: H3K27ac, H3K4me1, H3K4me3. The final panel shows gene locations. The red vertical line shows the location of the bait fragment

**Fig. 5:**
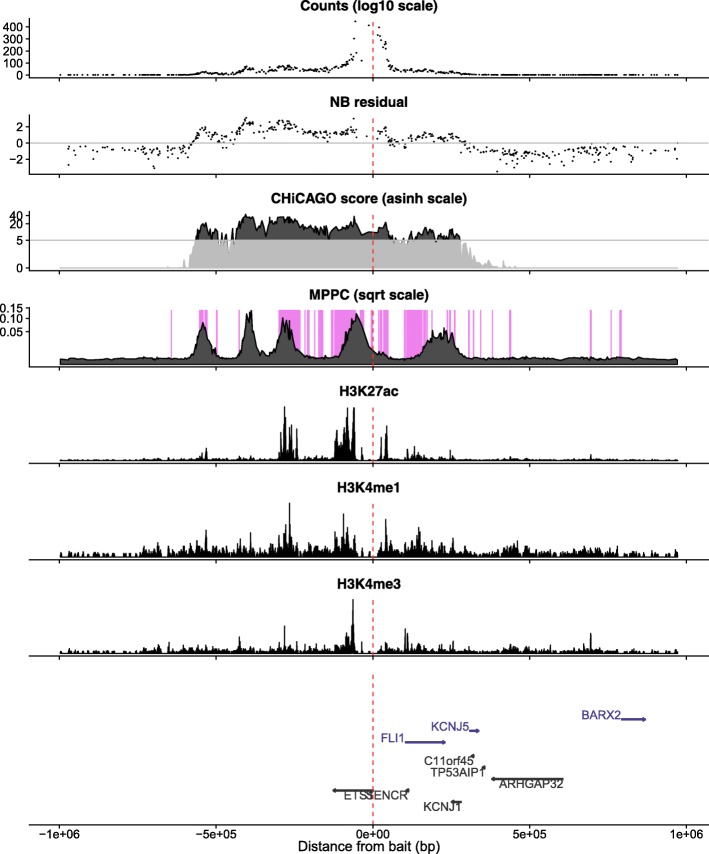
Illustrative example of analysis in the region of the *ETS1* promoter in activated CD4 ^+^ T cells. The top three panels show raw counts, adjusted residuals, CHiCAGO scores, and MPPC. The MPPC is overlaid on shading highlighting regions of active chromatin derived by aggregating states from CHROMHMM analysis ChIP-seq data from the same cell type, as previously published [7]. Note that the long run of fragments around and to the left of the promoter with high CHiCAGO scores have MPPC profiles which visually cluster into five groups, all of which correspond to regions with active chromatin marks (i.e. they overlie shaded regions). The next three panels show three examples of this CHiP-seq data: H3K27ac, H3K4me1, H3K4me3. The final panel shows gene locations. The red vertical line shows the location of the bait fragment

Finally, we illustrate these results by considering example baits with long runs of CHiCAGO-significant prey fragments. First, a bait on chromosome 2, which is annotated with both an antisense gene AC009505.2 and an alternative promoter of *NCK2*, is linked to long runs totalling 198 CHiCAGO-significant prey fragments which our model suggests can be explained by subsets of 7 fragments (Fig. 4). Visually, the MPPC profiles here also cluster into 7 groups. In particular, the stretch of fragments surrounding the promoter which are split by MPPC into 5 groups, each of which correspond to regions with active chromatin marks. Distinct clusters are not always visually distinguishable, of course. Figure 5 shows the analysis in the region of the *ETS1* gene promoter. Here, runs totalling 218 prey fragments have CHiCAGO scores >5 which our joint analysis suggests may be explained by a subset of 7 fragments. Visually, 6 clusters can be discerned in the MPPC profiles, all in similar locations to peaks in H3K27ac, but the sum of MPPC over fragments in the third cluster from the left is 1.91, suggesting this cluster may harbour two direct contacts, although their distinct locations are not clearly separable.

The MPPC is a continuous measure from 0 to 1, rather than a yes/no discriminator. We evaluated its utility according to its correspondence with characteristics expected in direct contacts.

## Discussion

Our results support our hypothesis that long runs of prey fragments with high counts in CHi-C data can result from a smaller number of direct contacts together with collateral signal at their neighbours. This suggests that efforts to jointly model the pattern of counts across multiple fragments have potential to distinguish those direct contacts. Joint modelling to improve resolution is already used to fine map genetic causal variants in genome-wide association studies (GWAS). It is accepted in GWAS that the p value corresponding to a test of association between a single genetic variant and some phenotype should be interpreted in the context of the *p* values of its neighbours, either by highlighting the variant in the region with the smallest p value, or by fitting a variable selection model to find a sparse subset of variants which could explain the association signals across the region. The primary difference between our CHi-C model and the class of GWAS fine mapping models that also fit the association statistics directly (e.g. PAINTOR, [18]) is that the decay of GWAS association signals across genetic variants has been established to relate to the linkage disequilibrium or correlation between those variants within the population, while our model assumes an exponential decay specified by a single parameter *ω*. We chose *ω* by considering a range of values and choosing that which produced residuals without obvious autocorrelation. This meant we could parallelise our analysis, considering each bait independently, but different values of *ω* would produce different results. Future work will explore whether it is computationally feasible to specify *ω* within a hierarchical framework that considers multiple baits simultaneously. In addition, we intend to investigate whether these ideas – using information from sets of proximal locations in a joint model to make inference about each individual location – could be adapted to other techniques used to call DNA contacts, such as next generation Capture-C [19], ChIA-PET [20] and Hi-ChIP [21], although different decay functions might be required.

In addition to jointly modelling the signal across multiple fragments, our proposed model contrasts to previous efforts to analyse CHi-C data by producing a Bayesian measure of confidence in the location of a direct contact - the MPPC. Both the MPPC and the CHiCAGO score decay with distance from bait, emphasising that short range contacts predominate, at least within the set of contacts detectable through CHi-C. There is, though, a notable difference between the rates of decay (Additional file 2: Figure S3). This reflects the deliberate choice of the CHiCAGO authors to weight *p* values such that more distant interactions were less likely to be called significant. We chose not to adopt any distant-dependent prior as our intention was to fine map contacts already called by a method that incorporates this distance penalty, such as CHiCAGO, and we did not wish to doubly penalise distant contacts. However, it is possible that adopting such a prior would lead to improved inference were our joint model to be applied alone. We also note that while we have broadly followed the CHiCAGO pre-processing approach here so that our results can be considered a fine mapping layer on top of a standard CHiCAGO analysis, other pre-processing would be possible. For instance, for bait fragments near a TAD boundary, the counts are likely to have a different distribution (fewer counts) towards the boundary rather than towards the centre of the TAD. While our approach is agnostic to the location of TAD boundaries, other methods such as PeakC [22] explicitly account for asymmetrically distributed counts around bait and could be used as alternate models to generate standardised residuals, followed by peaky fine mapping.

We argue that our joint analysis of neighbouring prey fragments adds a further useful dimension to the analysis of CHi-C data, with a CHiCAGO score reflecting the (distance from bait-adjusted) evidence for there being any contacts in a neighbourhood, and the MPPC reflecting the expected number and the uncertainty in the precise location of direct contacts. Other advantages of adopting a Bayesian framework include the ability to extend the model to include not just bait-prey distance, but other prior information on the likelihood of direct contacts. This would enable, for example, information from previous experiments in related cell types to inform future analyses.

## Conclusions

We have proposed a new model for calling direct contacts from CHi-C data that, in contrast to existing fragment-by-fragment analysis methods, exploits information from each prey’s neighbouring fragments. Our joint model identifies prey fragments with biological characteristics that would be expected at sites of direct contact, such as an active chromatin state when they contact promoters. We have shown this information is largely complementary to that produced by the per-fragment method, CHiCAGO. Combining inference across these two approaches is more stringent – a prey fragment needs to simultaneously have a higher count than expected **and** a supporting pattern among neighbouring fragments – and leads to improved resolution of direct contacts in CHi-C datasets.

## Methods

### Pre-processing of read count data

We first pre-process the read count data using similar methods to standard CHi-C analysis to produce residuals which have a standard normal distribution in the absence of interactions. The raw data for a CHi-C experiment takes the form of a sparse matrix of counts for pairs of baits and preys. In practice, most entries in this matrix are zero, and analysis focuses on modelling the counts at preys that are within some linear genomic distance of each bait. Statistical inference of contacts is based on a two step approach. First, counts are modelled to adjust for systematic effects such as distance between bait and prey, and capture efficiency using either negative binomial (NB) regression [9] or a convolution of NB and Poisson regression, to model biological and technical noise separately [6]. Second, a decision is taken to call contacts based on comparing observed counts to those expected under this empirical model estimated under a null hypothesis of no true interactions, either using raw *p* values [9] or *p* values weighted to allow for the complication that we expect to find more interactions among fragments proximal to the bait, but test many more long distance pairs. We wished to use the first part of this procedure to account for the systematic effects in the data, and generate standardised residuals (that is, residuals with unit variance) for input into our proposed joint model.

The CHi-C data from CD4 ^+^ cells that we propose to use for this study have previously been processed by the HiCUP pipeline [23] and CHiCAGO [6] as described in [8]. We noted that the technical noise component had a generally small contribution compared to the biological noise (Additional file 2: Figure S8). We therefore applied NB regression alone to the raw counts using standard software to generate these standardised residuals, which we call NB residuals in the text below. This allowed us to add additional covariates to the regression which we found provided small improvements to the model fit. Besides the distance between an interaction’s bait and prey fragments, and whether both fragments in a putative contact were baited, we used the length of both fragments, as well as transchromosomal bait activity, as covariates. Assuming that transchromosomal contacts are equally rare across baits [24], the latter is a proxy for enrichment and capture biases. To account for the difference in the number of possible transchromosomal interaction sites between baits on different chromosomes, transchromosomal bait activity is defined for each bait as the residual following from the regression of the sum of its transchromosomal counts against its chromosome number. We used the R package GAMLSS to fit zero-truncated NB models to counts for each pair of bait (*b*) and prey (*p*) within ten distance bins (Additional file 2: Table S3). Assuming most bait-prey fragment pairs do not make direct contacts, the null model can be parametrized using the full dataset. We used normalized, randomized quantile residuals [25] as the input for our joint model. A comparison between the predicted fits from CHiCAGO and our NB models applied to the same data showed a good correspondence (Additional file 2: Figure S9).

### Priors on model parameters

For each bait *b*, the non-zero strengths were assigned independent normal priors centered on 0, with a common variance $\sigma ^{2}_{\beta }$:

4 $$  \left[ \beta_{bq}|\gamma_{bq}=1 \right] \sim N\left(0, \sigma^{2}_{\beta}\right) \text{ for} \,p,\ldots,F_{b}  $$

Rather than fixing *σ*_*β*_, which controls the magnitude of interaction strengths supported by the model, and therefore can have an important impact on the efficiency of the algorithm, we use a flexible hyper-prior allowing adaption to the data. Specifically, we placed a weakly informative Uniform(0.01,2) hyper-prior on *σ*_*β*_. The median, *σ*_*β*_=1, corresponds to 95% support for interaction strengths up to a plausible 1.96. However, this hyper-prior equally supports much smaller values of *σ*_*β*_, as well as values up to the maximum of 2, corresponding to support for interaction strengths as large as 8 — marginally larger than any individual NB residual we observed (Additional file 2: Figure S1).

Our model selection framework is completed by specifying a prior for each *γ*_*bp*_. To avoid problems of over-fitting from simultaneous estimation of too many interaction parameters, and because we believe a priori that direct contacts only exist at a small proportion of prey fragments, our prior on *γ*_*bp*_ is designed to encourage a “sparse” selection of interactions. To this end we first define *θ*_*b*_, the expected proportion of prey fragments which contact *b*, i.e. $\frac {1}{F_{b}}\sum _{q} \gamma _{bq}$, which has prior distribution

5 $$  \theta_{b} \sim Beta(1, F_{b}).  $$

Conditional on *θ*_*b*_, each *γ*_*bq*_ is then *i.i.d.* Bernoulli(*θ*_*b*_). This prior has two attractive properties. First, the marginal prior odds that a particular fragment interacts with *b* is 1/*F*_*b*_, and therefore decreases with the total number of fragments considered. Meanwhile, the prior odds for there being no interactions is a constant 0.5 for every bait. This setup provides an intrinsic multiplicity correction for the number of fragments in each bait, and allows fair comparison of inference across baits, due to the common prior on the null model [26]. Note too that this corresponds to a very small prior odds of interaction for each individual fragment, since *F*_*b*_ is usually in the order of 3000, and thereby encourages the exploration of sparse models.

### Model fitting via reversible jump MCMC

For bait *b*, the Reversible Jump MCMC [27] sampling scheme starts at an initial set of interactions, ***γ***_*b*_, and corresponding strengths, ***β***_*b*_, denoted ***γ***_*b*_(0) and ***β***_*b*_(0) respectively. Bold symbols are used to denote that these are vectors across all fragments in the neighbourhood of bait *b*. To sample the next set of interactions and strengths, which we denote ***γ***_*b*_(1) and ***β***_*b*_(1), we propose moving from the current state to another combination of interactions and/or set of strengths, ***γ***_*b*_∗ and $\boldsymbol \beta _{b}^{*}$, by using a proposal function $g(\boldsymbol \gamma _{b}*, \boldsymbol \beta _{b}^{*} | \boldsymbol \gamma _{b}, \boldsymbol \beta _{b})$. We then accept these proposed values as the next sample with probability equal to the Reversible Jump Metropolis-Hastings ratio:

$${\begin{aligned} MHR = \frac{P\left(\boldsymbol Y_{b} | \boldsymbol\gamma_{b}*,\boldsymbol\beta_{b}^{*}\right) \pi\left(\boldsymbol\beta_{b}^{*} | \boldsymbol\gamma_{b}*\right) \pi\left(\boldsymbol\gamma_{b}*\right)} {P\left(\boldsymbol Y_{b} | \boldsymbol\gamma_{b},\boldsymbol \beta_{b}\right) \pi\left(\boldsymbol \beta_{b} | \boldsymbol\gamma_{b}\right) p\left(\boldsymbol\gamma_{b}\right)} \times \frac{g\left(\boldsymbol\gamma_{b}, \boldsymbol\beta_{b} | \boldsymbol\gamma_{b}*, \boldsymbol\beta_{b}^{*}\right)}{g\left(\boldsymbol\gamma_{b}*, \boldsymbol\beta_{b}^{*} | \boldsymbol\gamma_{b}, \boldsymbol\beta_{b}\right)} \end{aligned}} $$

where ***Y***_*b*_ are the residuals of all fragments captured for bait *b*, *P*(***Y***_*b*_|.) is the model described by (1) and (3). *π*(***β***_*b*_|***γ***_*b*_) is the prior distribution of the strengths defined in (4) conditional on the corresponding interactions being included in the model. *p*(***γ***_*b*_) is the model space prior defined in (5). Therefore the proposed combination of interactions and new strength values are accepted with a probability proportional to their likelihood and prior. If this new set of values is accepted, the proposed set is accepted as ***γ***_*b*_(1) and ***β***_*b*_(1); otherwise, the sample value remains equal to the current sample value, i.e., ***γ***_*b*_(1)=***γ***_*b*_(0) and ***β***_*b*_(1)=***β***_*b*_(0). It can be shown that this produces a sequence of parameter samples that converge to the required posterior distribution [27]. We used the Reversible Jump MCMC implementation in R2BGLiMS (https://github.com/pjnewcombe/R2BGLiMS, [16]), to fit the model described above to each bait in turn. Because our aim was to fit this model to 28,214 baits, we took some time to define a strategy for thinning these samples in order to perform reliable posterior inference on *γ*_*b*_ while minimising the computational burden (Additional file 1). This led us to run R2BGLiMS sampling 5000 models per chain, at a density of 1 per 1000 iterations, with no burn-in. We ran two parallel chains for each bait, and checked convergence between MPPC derived from each chain. If this was <0.75, we ran a further 5000 samples to improve convergence. Autocorrelation plots were also used to evaluate model space exploration for individual baits (Additional file 2: Figure S10).

### Assessing relationship of CHiCAGO scores and MPPC to outcome measures

CHiCAGO scores are non-negative real numbers, and are typically asinh transformed for presentation or downstream inference, to prevent over-leverage of points in the extreme right of the distribution [8]. In constrast, MPPC lies between 0 and 1, although rarely reaches 1 in practice. We found MPPC were generally positively correlated with CHiCAGO scores, with the relationship closest to linear when sqrt(MPPC) was compared to asinh(CHiCAGO) (Additional file 2: Figure S4). We therefore use a square root transform in following analyses to perform a fair comparison with CHiCAGO scores.

We defined the following four outcome measures:

- **Promoter: match to** [17] For validation with external promoter-enhancer networks, we used the positions given in http://yiplab.cse.cuhk.edu.hk/jeme/encoderoadmap_lasso/encoderoadmap_lasso.34.csv (accessed 2017/09/11). We used GenomicRanges to identifying bait-prey fragment pairs which overlapped the paired co-ordinates given in this file, and set a binary outcome 1 if such an overlap was found and 0 otherwise. Analyses of this measure were restricted to prey fragments within 200 kb of the bait, because 95% of these reported links were within that range.
- **Promoter: link to active chromatin** These cells had previously been assayed by ChIP-seq, and a 15 state CHROMHMM model fitted. 8 of these states showed characteristics of “active chromatin” and we combined these into a binary measure for active or inactive chromatin [7]. We used these results to quantify the overlap, for each prey fragment, with regions of active chromatin. For the most part (∼90*%*), a fragment showed complete overlap or lack of overlap with active chromatin regions, in which case the outcome measures was set to 1 or 0 respectively. To allow logistic regression of this mainly binary outcome, the observations with fractional overlap were set to missing for analysis.
- **Validation: overlap baited promoter** For a measure of promoter overlap, we used the binary indicator of whether a prey fragment in the validation experiment had also been baited in the promoter experiment.
- **Validation: expression at linked promoter** Given evidence that recruitment of prey fragments is associated with increased expression of the baited gene [7], we expected that, amongst prey that did correspond to a baited promoter in the promoter capture experiment, the level of expression of the target gene should be higher when there was a direct contact. RNA-seq has previously been used to quantify transcription in these cells, and we used the expression of the target gene (log_2_(count + 1)) as an outcome measure in linear regression. Analyses of this measure were restricted to bait-prey pairs where the prey corresponded to a gene promoter.

Because each prey fragment is represented multiple times (with different baits), we assessed the relationship between asinh-transformed CHiCAGO scores and sqrt-transformed MPPC with each outcome measure using robust clustered linear or logistic regression implemented in the R library rms (https://cran.r-project.org/web/packages/rms/), clustering on the prey fragment.

## Additional files

Additional file 1 Supplementary Note. Detailed description of the statistical model underlying peaky, and parameter choices. (PDF 1650 kb)

Additional file 2 Supplementary Figures and Tables. (PDF 4180 kb)

Additional file 3 Supplementary Data. The processed CD4 and macrophage data are in supplementary data supp-data.tgz. This gzipped archive contains .csv files (suppdata-*.csv), one for each experiment, with bait and prey *hind*III fragment IDs together with columns:
∙ N read count
∙ residual NB residual
∙ mppc MPPC
∙ beta.post posterior expectation of *β*
∙ chicago CHiCAGO score (only for CD4 cells)
and two annotation files:
∙hind-positions.csv gives the chromosome co-ordinates of each *hind*III fragment
∙bait2gene.csv gives the links between promoter baits and annotated genes, using ensembl 75. (ZIP 673,425 kb)
