# Supplementary material for: Fine mapping chromatin contacts in capture Hi-C data
Source: BMC Genomics. 2019 Jan 23;20:77. doi: 10.1186/s12864-018-5314-5 (PMC6343296; doi:10.1186/s12864-018-5314-5)
Supplement: Supplementary file 1 — Supplementary Note. Detailed description of the statistical model underlying peaky, and parameter choices. (PDF 1650 kb) [file 12864_2018_5314_MOESM1_ESM.pdf]

Fine mapping of interactions from capture Hi-C data: supplementary note

Christiaan Eijsbouts   Paul Newcombe   Chris Wallace

November 21, 2018

1 Choice of distance function and omega

In choosing the form and parameter values for the decay function in (2), we considered three different functions:

$$\delta(p,q;\beta_{bq},\omega) = \begin{cases} \beta_{bq} \times (d(p,q)+1)^{\omega} & \text{(A)} \\ (\beta_{bq})^{-d(p,q)*\omega} & \text{(B)} \\ \beta_{bq} \times \exp -d(p,q) * \omega & \text{(C)} \end{cases}$$

where $d(p,q)$ is the absolute distance between the midpoints of fragments p and q.

We empirically determined the best function by fitting our model (3) with different choices of $\delta(p,q)$ to a subset of input data and considering the properties of the residuals from this model. Statistically, these should be i.i.d. with mean 0 and unit variance. To assess this, a consensus fit was constructed for each bait b using the posterior expectations of β_{bp}, $\hat{\beta}_{bp}$. The mean and variance of the residuals from these fitted models were calculated across overlapping sliding windows over 250 prey fragments, and subsequently aggregated. Residuals most closely resembling $N(0,1)$ were obtained for option (C) with $\omega = 10^{-4.7}$ (Figure 1). Finally, we confirmed that this value of ω produced similar distributions of residual mean and variance across all baits (Figures 2).

Code may be found in sliding_window.R and sliding_window_visualize.R at `https://github.com/chr1swallace/hic`.

2 Determining computationally efficient sampling whilst maintaining RJMCMC convergence

RJMCMC approximates a posterior distribution by stochastically sampling candidate models. How many candidate models we generate overall, and how we sample them, determines how accurately these samples reflect the posterior distribution and hence how reliable our inference of coefficient

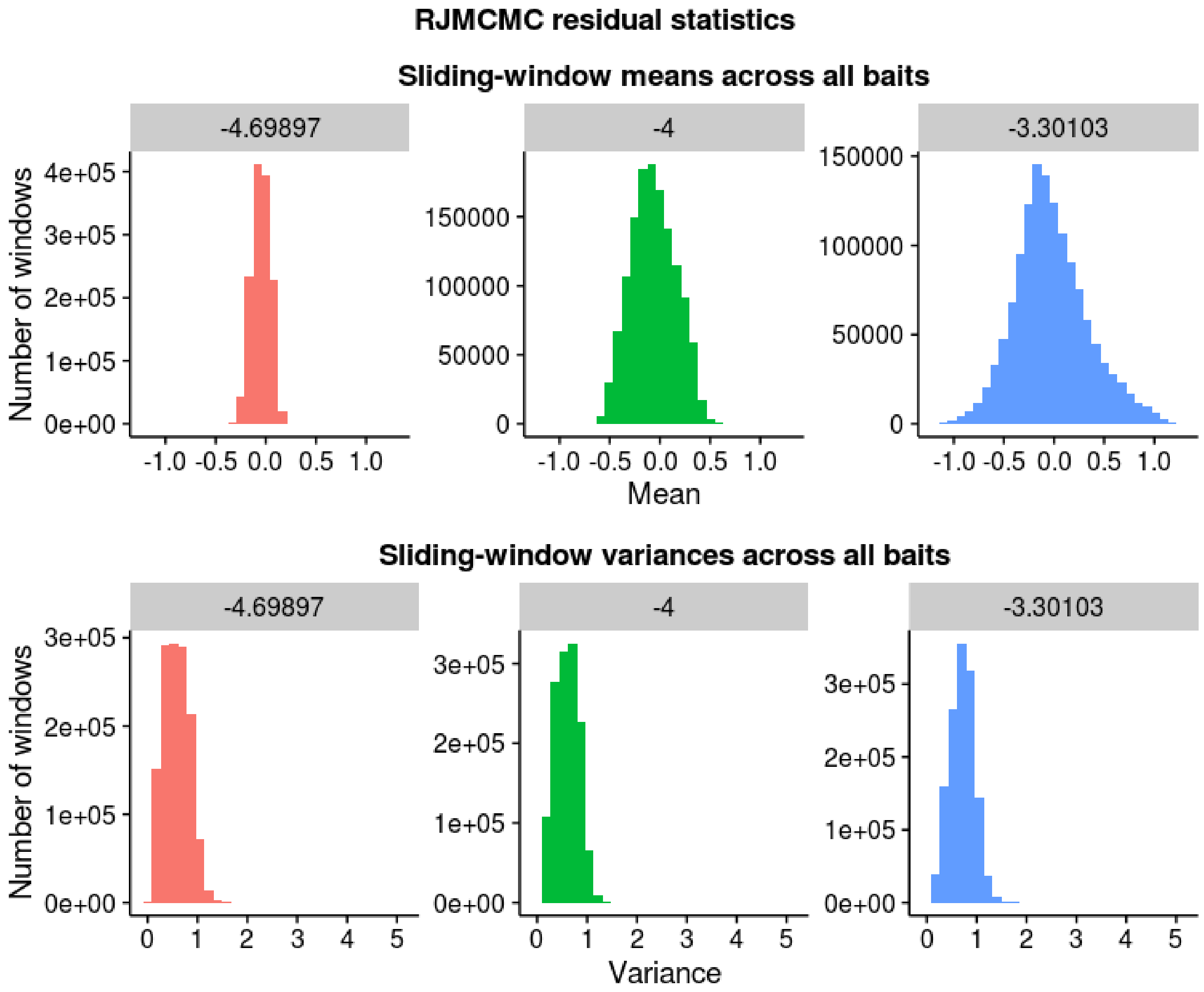

Figure 1: Sliding window means and variance of residuals from our joint model fitted using different values of ω. We chose the value of ω that gave means and variances closest to 0 and 1 respectively.

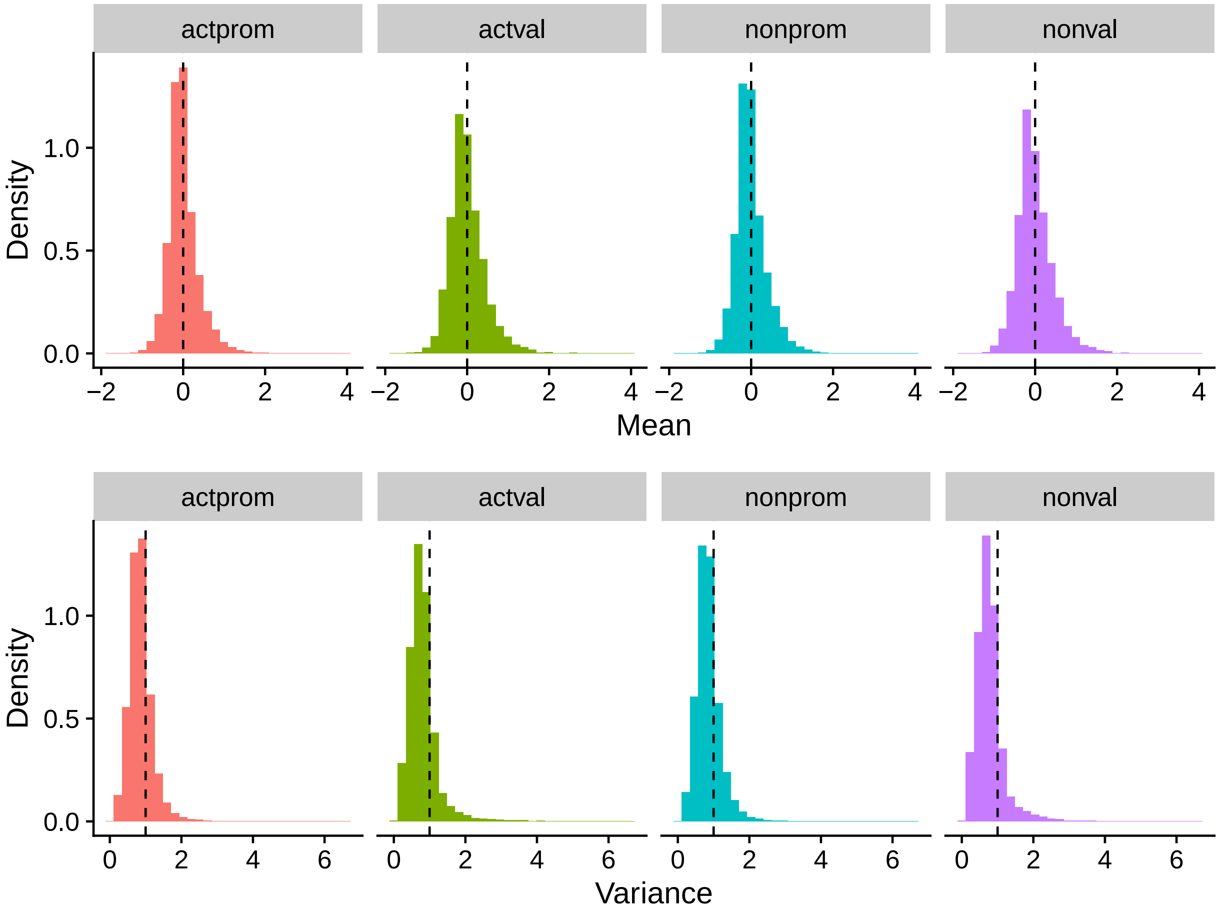

Figure 2: Distribution of mean and variance of residuals from our model, calculated within non-overlapping windows of 250 preys, are concentrated around the target values of mean=0 and variance=1. Each histrogram shows the distribution across all windows across all baits for each experiment.

values is. To find a balance between minimizing computational cost and maximizing the reliability of the results, we asked three questions:

1. How many models should we generate?
2. At which point do we start sampling?
3. What must the sampling density be?

First, we obtained "gold standard" MPPC values for a single, hand-picked bait (with a complex PCHi-C signal) by generating an extremely large number of models ($N = 20 \times 10^6$) and sampling an impractically large number of them ($n = 100,000$) from the last 10×10^6. The first 10×10^6 models are thereby considered to be biased by the initial candidate model –almost certainly a conservative overestimate– and discarded as "burn-in". We repeated this procedure 10 times with different random seeds, yielding 10 sets of gold standard MPPC values. To answer our questions above, we repeatedly resampled smaller subsets under different conditions from the 20×10^6 models (using the same 10 seeds) and gauged how similar the MPPC from these smaller samples were to the gold standard MPPC according to their correlation, ρ.

To answer 1., we fixed burn in at 10×10^6 and considered sampling from 200 to 5,000 models thereafter with a constant sampling density (sampling 1 in 2000). Sampling more models did consistently increase ρ, so we chose to sample 5,000 models total (Fig. 3). To answer 2, we fixed the sampling density (1 in 2000) and number of models (5,000) and varied the length of burn-in. We found ρ remains similar regardless of length of burn-in, so decided we could set burn-in length to 0 (Fig. 4). However, even with no burn-in, this type of sampling requires $2000 \times 5000 = 10 \times 10^6$ models to be generated in total. To answer 3, we fixed burn-in to 0, and number of sampled models to 5000, and varied the sampling density. We found that we could obtain $\rho > 0.75$ reducing the sampling density to the point where models for MPPC calculations where drawn from the first 5×10^6 models proposed (Fig. 5). Thus, we end up having to generate only 5×10^6 models in total, and sampling 5,000 of them with no burn-in.

In practice, we always run two parallel chains and confirm correlation of MPPC values produced is > 0.75, otherwise we extend the runs.

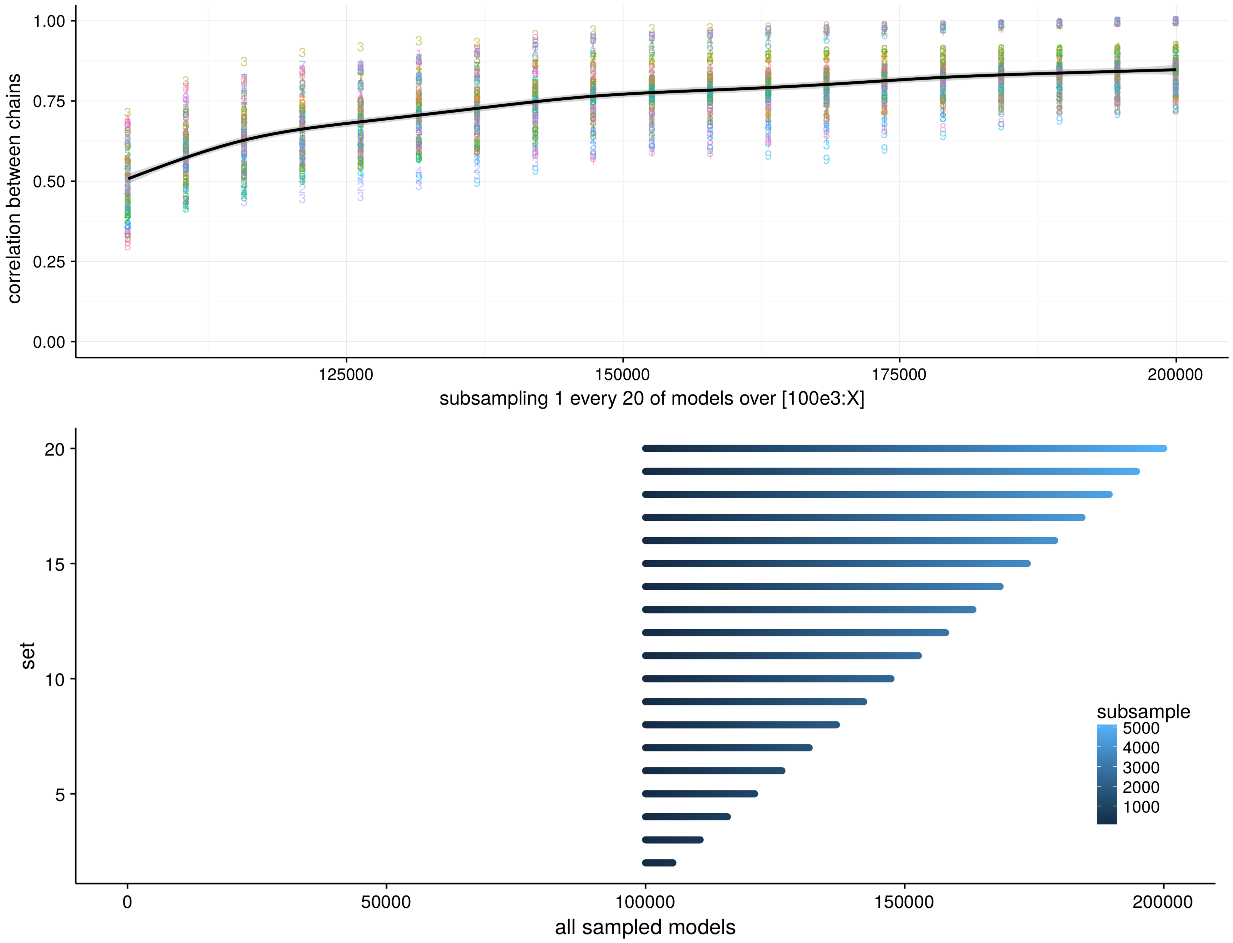

Figure 3: MPPC values obtained from a greater subset of models, sampled at a constant interval, correlate more closely with gold-standard MPPC values. As the number of models obtained before subsampling stops is raised (top, x-axis), the trend in correlation values between all possible pairs of gold-standard MPPC values (from 10 sets of 100, 000 models, distinguished by shapes) and MPPC values obtained through subsampling (of the same 10 sets, distinguished by colors) is upward. The bottom pane shows the subsampling strategy, with the selection of models (horizontal blue bar) increasing in size (brightness) while still excluding "burn-in" models (the first 100, 000). We decide to calculate MPPC values based on a minimum of 5000 models.

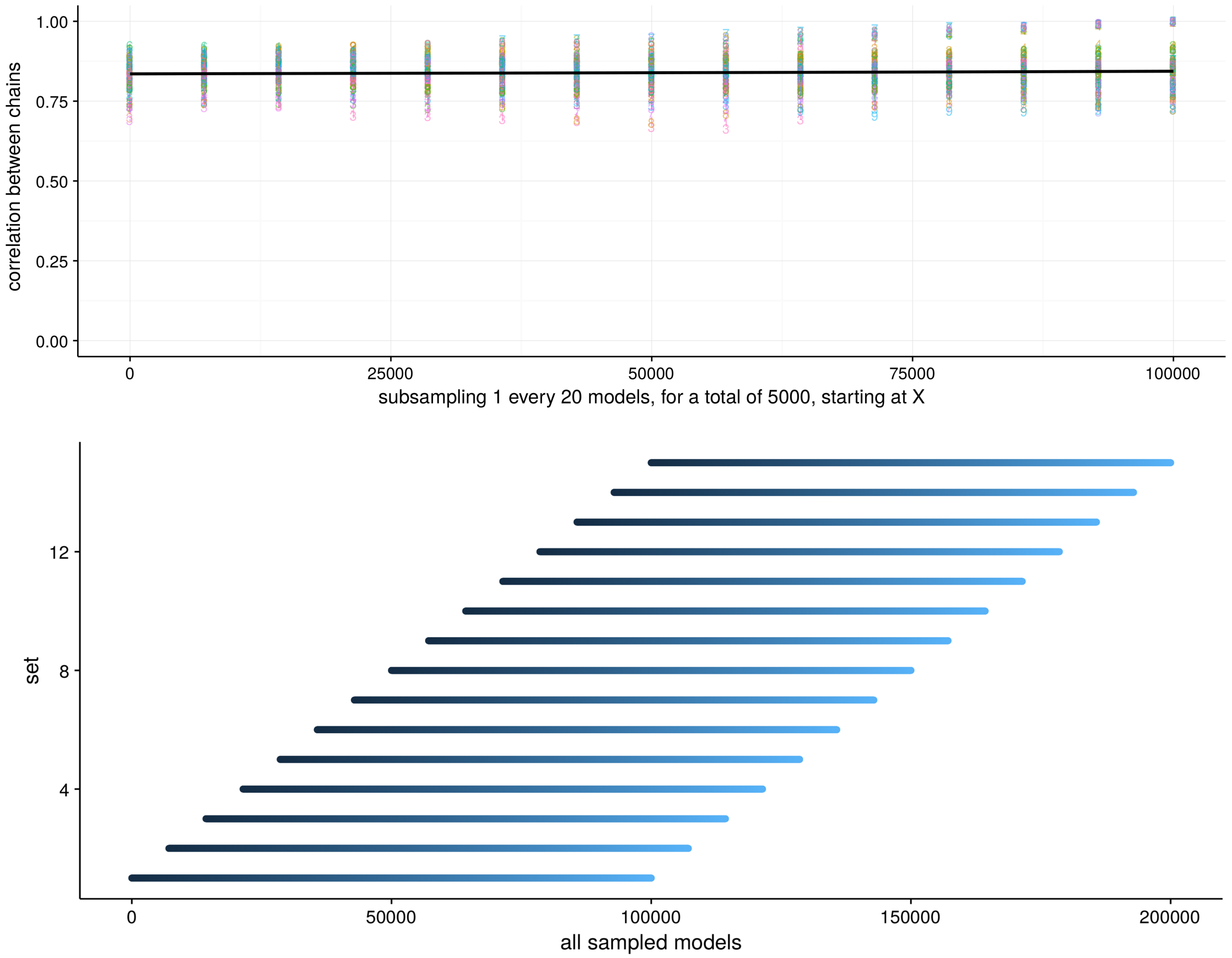

Figure 4: The sample of models from which MPPC values are calculated can be obtained from models proposed early on, given that subsampling of (an equal amount of) models proposed later does not yield MPPC values (10 sets, marked by colors) that correlate more closely with gold-standard MPPC values (10 sets, marked by shapes). The bottom pane shows the subsampling strategy, with equally-sized (same brightness) selections of $5,000$ models (horizontal blue bar) being subsampled from those proposed early or late (left and right on the x-axis, respectively) during the MCMC procedure. We decide that no "burn-in" phase must be waited out until sampling can begin.

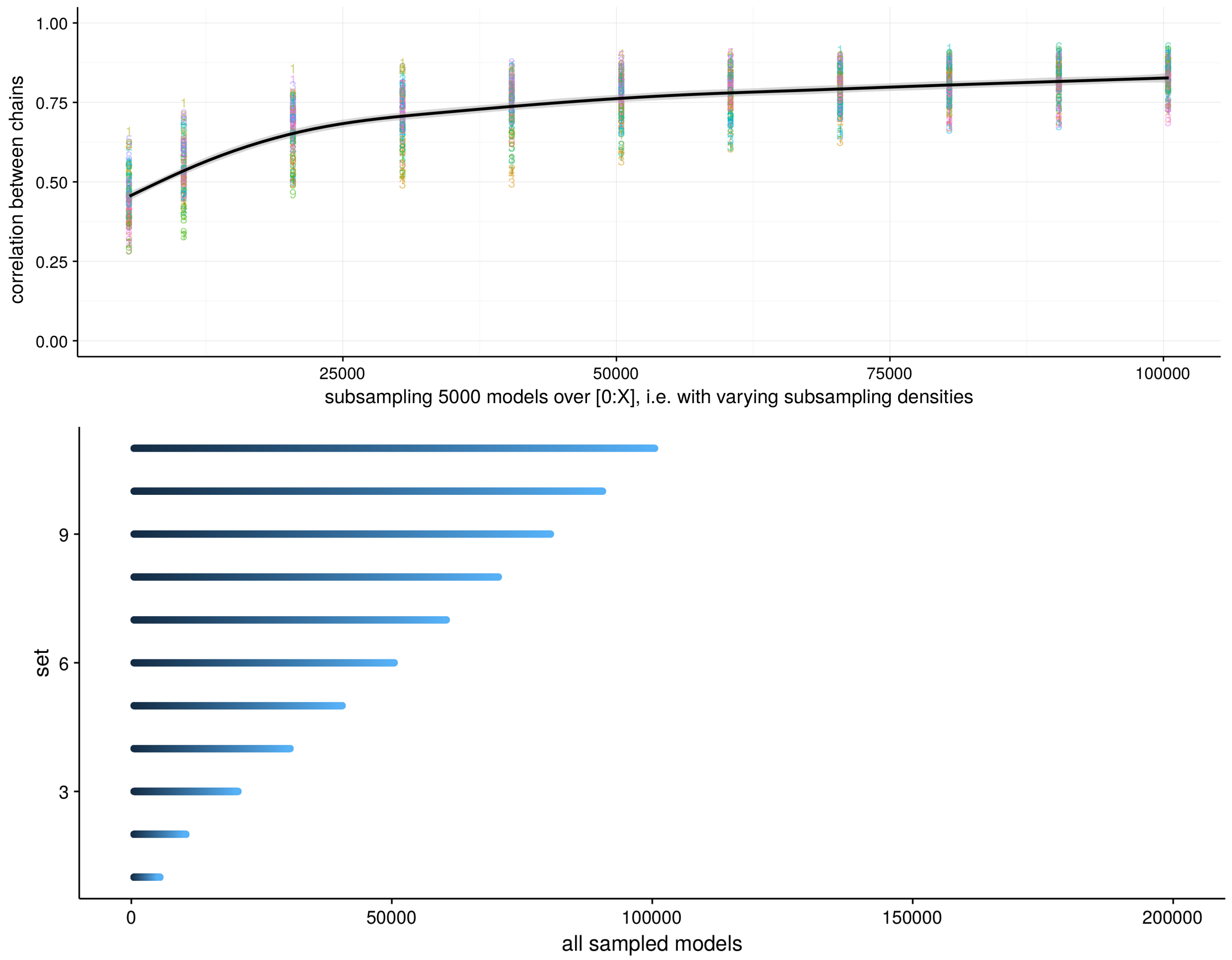

Figure 5: Decreasing the rate at which models are sampled increases the correlation between MPPC values obtained from those models (10 sets, marked by colors) and gold-standard MPPC values (10 sets, marked by shapes). When we begin subsampling immediately, increasing the range of proposed models from which a subsample of a given size (5000) is taken (bottom pane, blue bars representing model selections extend further but are ultimately equally bright) is helpful. We choose the sampling density such that we sample from a range of models extending to the 50,000th in this example, originally generated after 5 million proposals.
