# Supplementary material for: Fine mapping chromatin contacts in capture Hi-C data
Source: BMC Genomics. 2019 Jan 23;20:77. doi: 10.1186/s12864-018-5314-5 (PMC6343296; doi:10.1186/s12864-018-5314-5)
Supplement: Supplementary file 2 — Supplementary Figures and Tables. (PDF 4180 kb) [file 12864_2018_5314_MOESM2_ESM.pdf]

Figure S1: **Standardised residuals obtained from the NB regression model** QQ plots show that negative residuals and the lower 90 percentile of the positive residuals generally follow a standard normal distribution. Deviation from the standard normal (grey line) occurs at quantiles above 2, the top 10% of positive residuals.

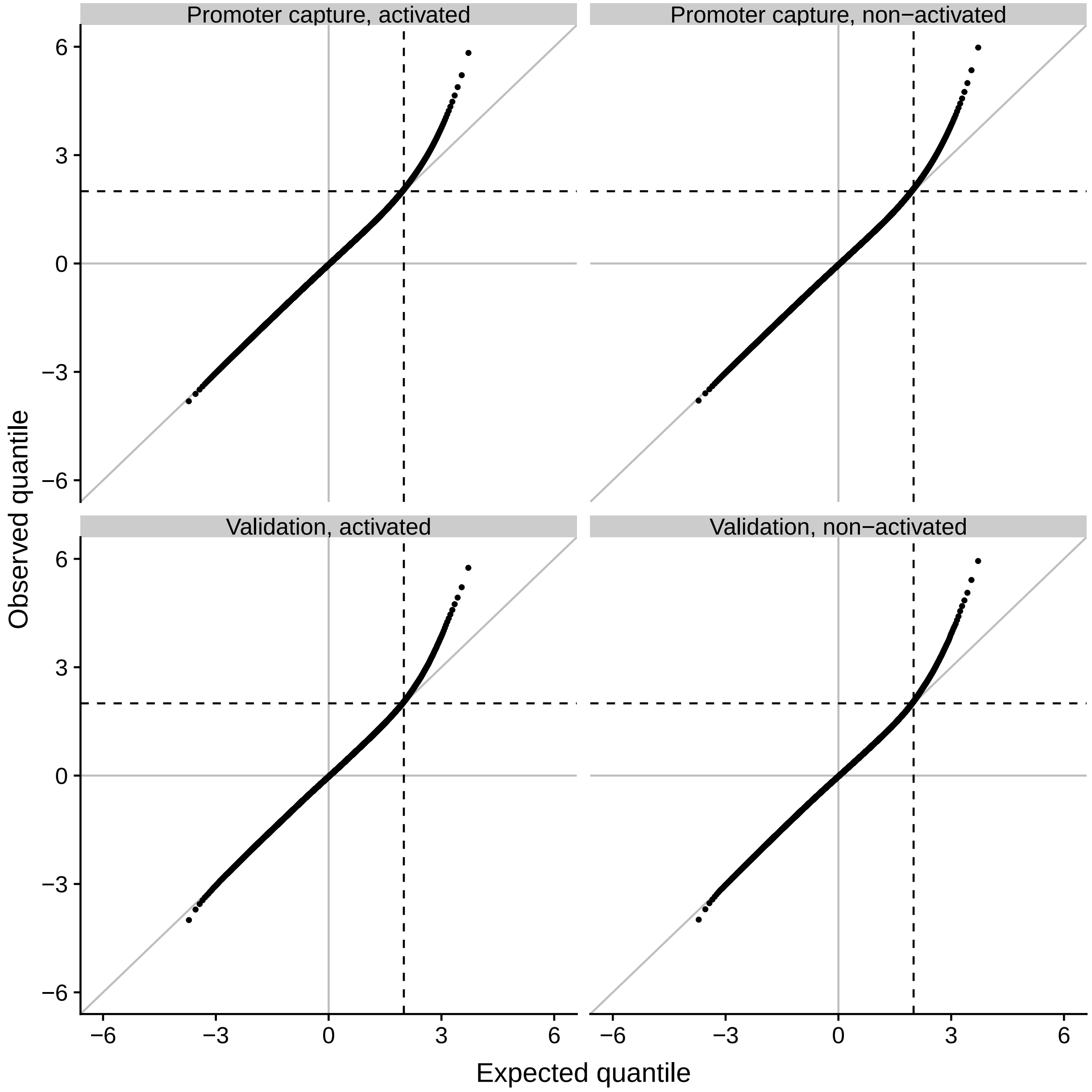

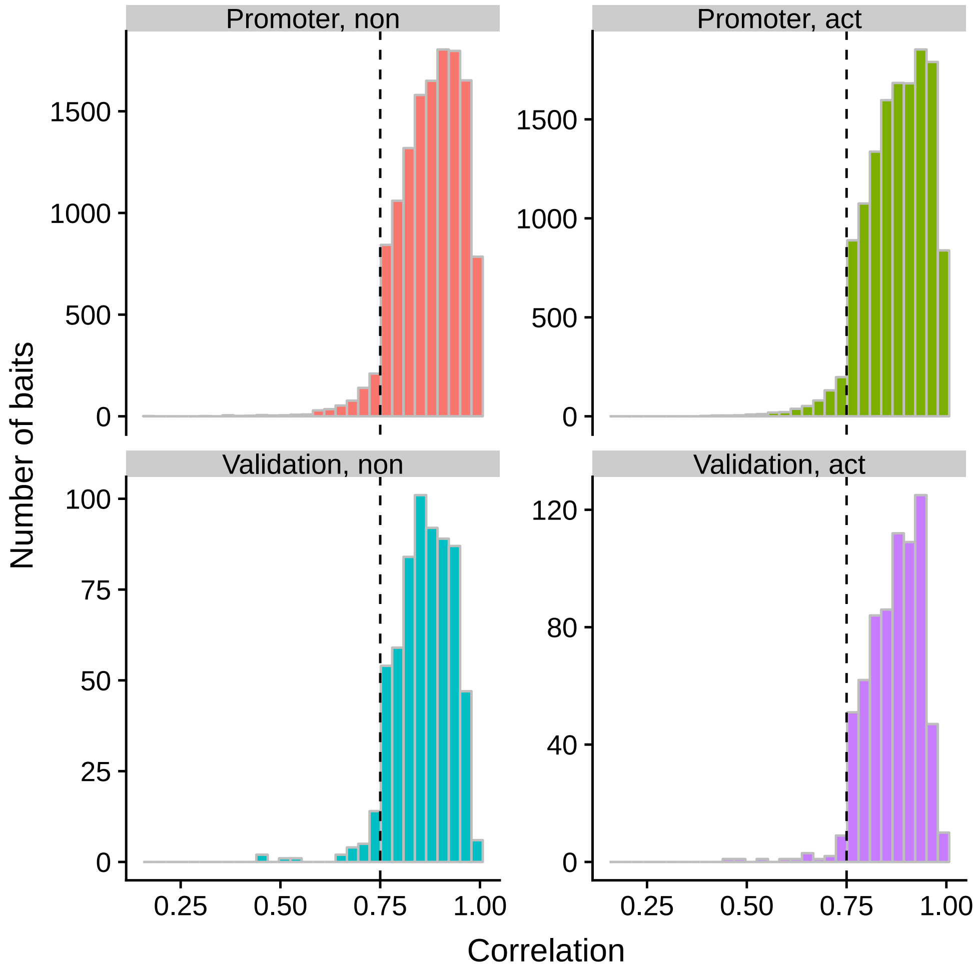


Figure S2: Distribution of correlation between MPCC from two parallel chains of RJMCMC run for each bait

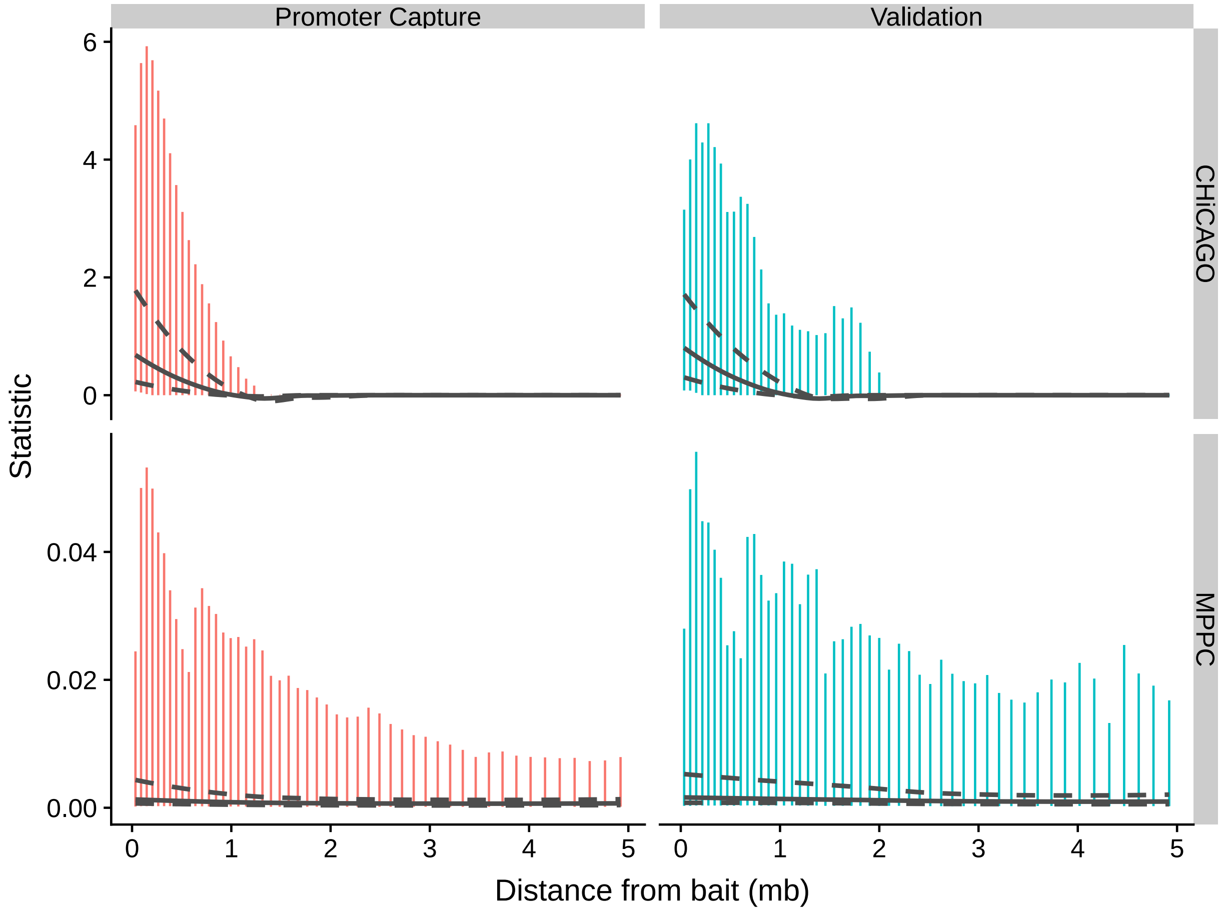


Figure S3: Decay of CHiCAGO scores and MPPC with distance from bait. The solid line shows the median value, the dashed lines the 25th and 75th centiles, and the limits of the vertical lines the 5th and 95th centiles.

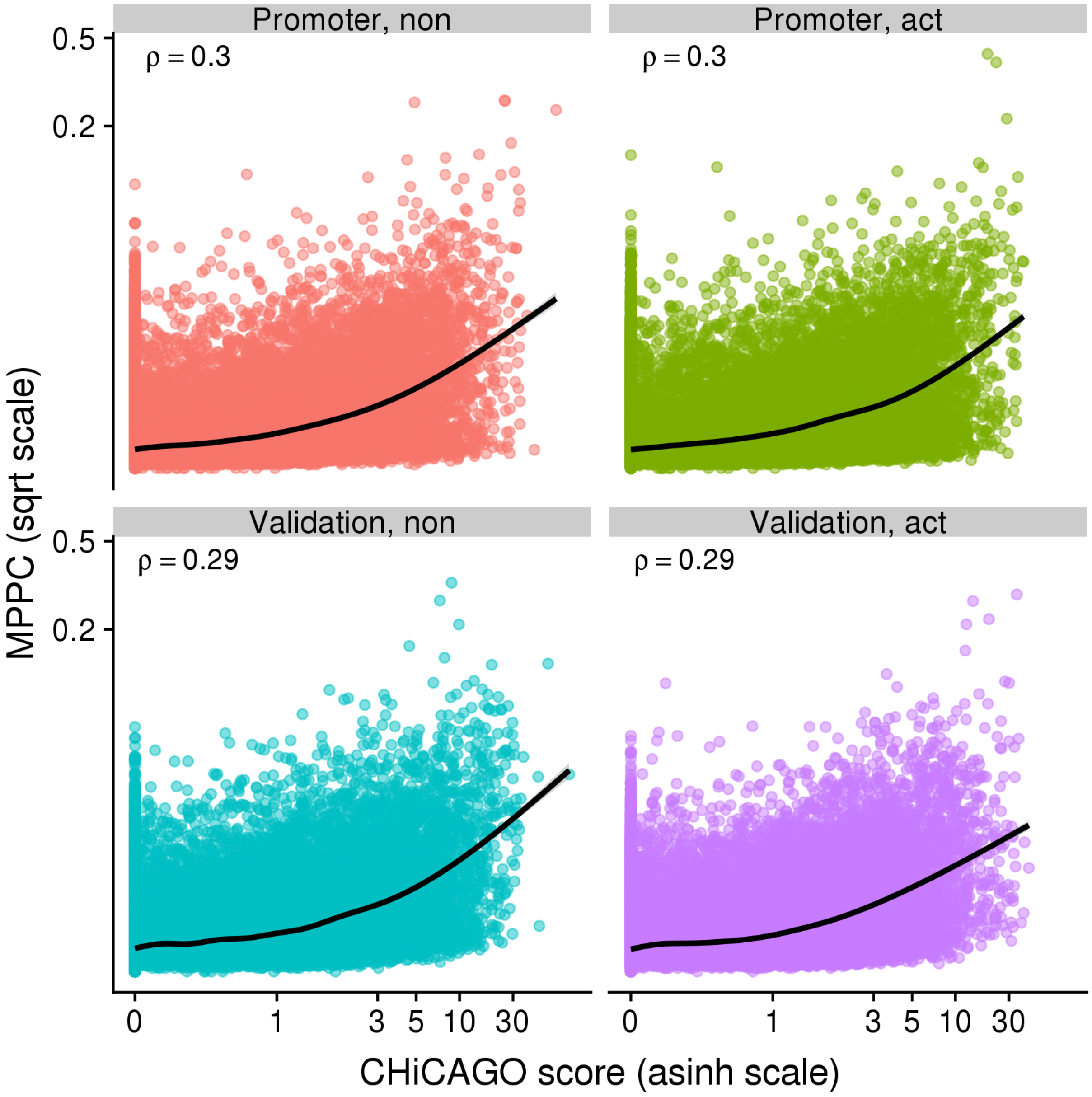


Figure S4: Comparison of CHiCAGO scores and MPPC for all potential bait-prey pairs considered for each experiment. Spearman's $\rho$ is given in the top right of each sub figure and the black line shows a loess smooth.

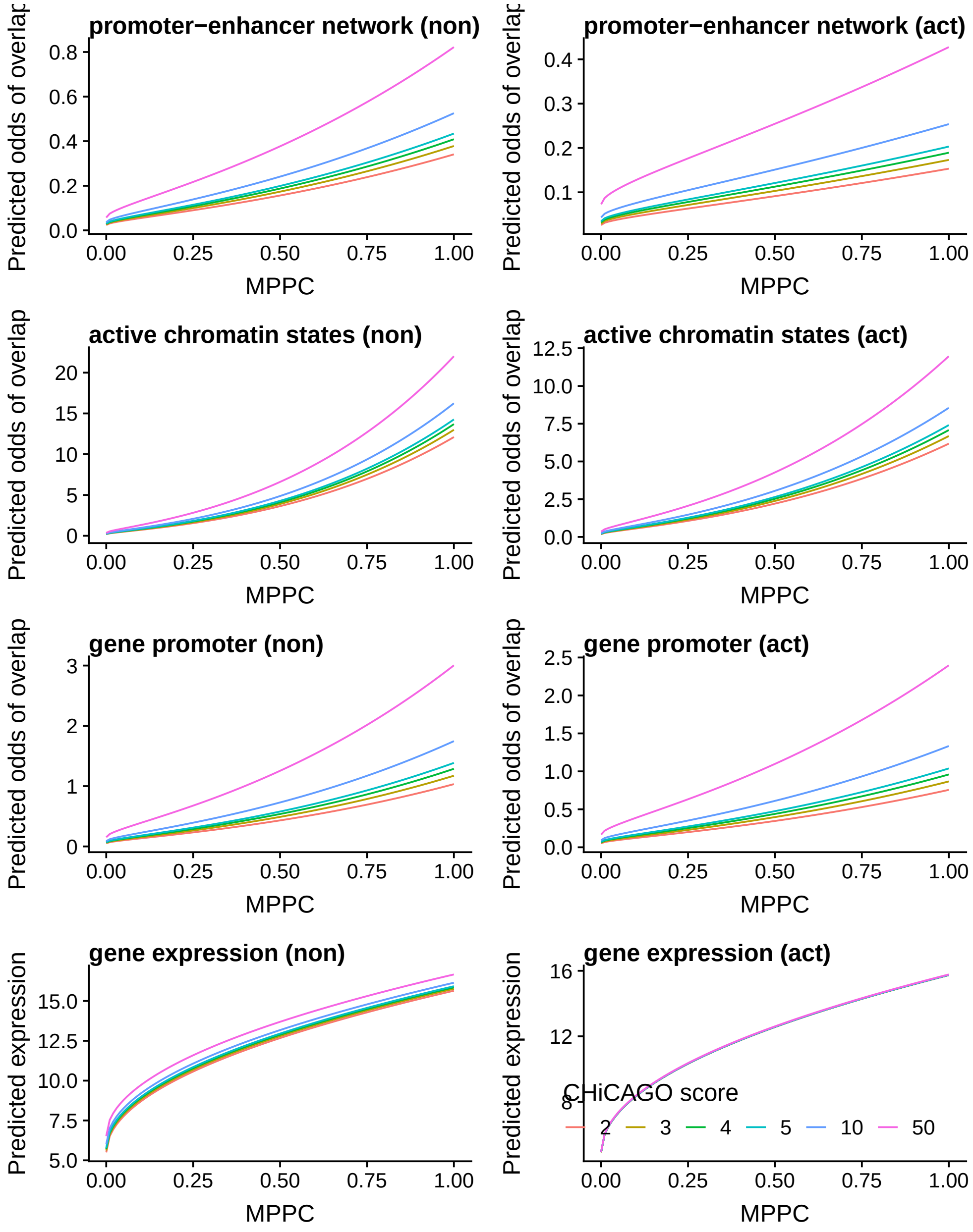


Figure S5: The prevalence of expected biological characteristics at contact sites increases with CHiCAGO's and Peaky's strength of evidence for a contact in two separate datasets. Fitted values from regression models of four outcome measures in our two datasets in non-activated (left column) and activated (right column) $CD4^+$ T cells. Row 1: promoter capture, odds that prey fragment overlaps enhancer in published T cell promoter-enhancer network [17]. Row 2: promoter capture, odds that prey fragment overlaps regions of active chromatin called by CHROMHMM of the same cells [9]. Row 3: validation, odds that prey fragment was baited in the promoter capture. Row 4: validation, average gene expression (counts, $\log_2$ scale) of gene associated with the baited promoter in RNA-seq analysis of the same cell type [9]. Predictors are CHiCAGO score (asinh transformed) and MPPC (sqrt transformed).

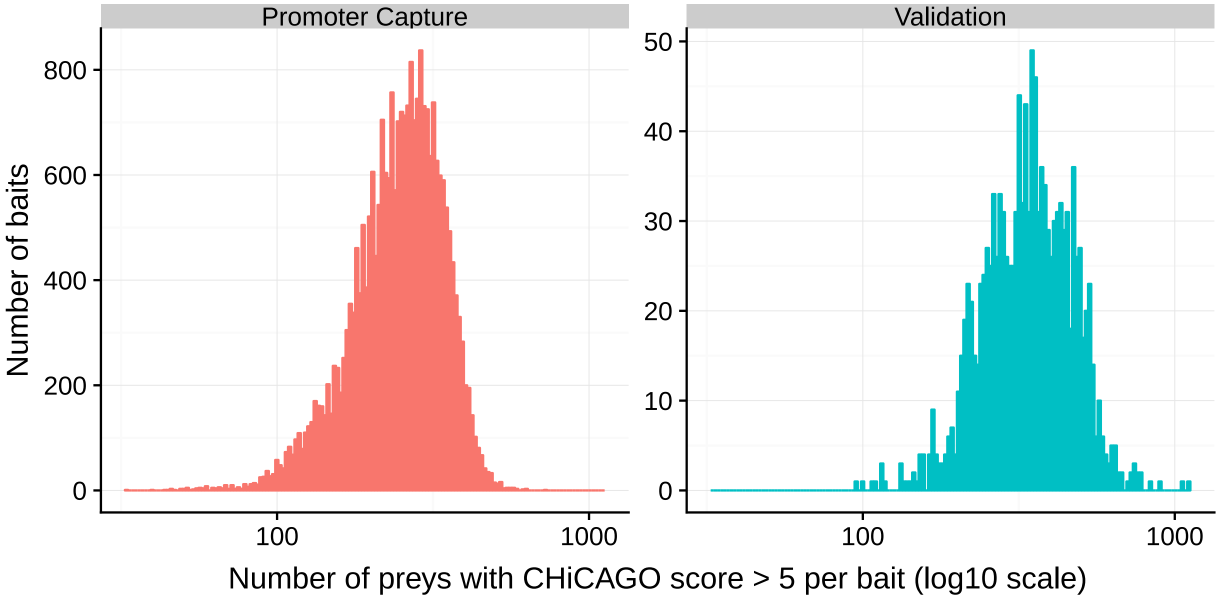


Figure S6: Distribution of number of contacts called significant (score $>5$) by CHiCAGO for each bait that had at least one CHiCAGO significant contact. Results from activated and non-activated cells were very similar and are combined into a single panel.

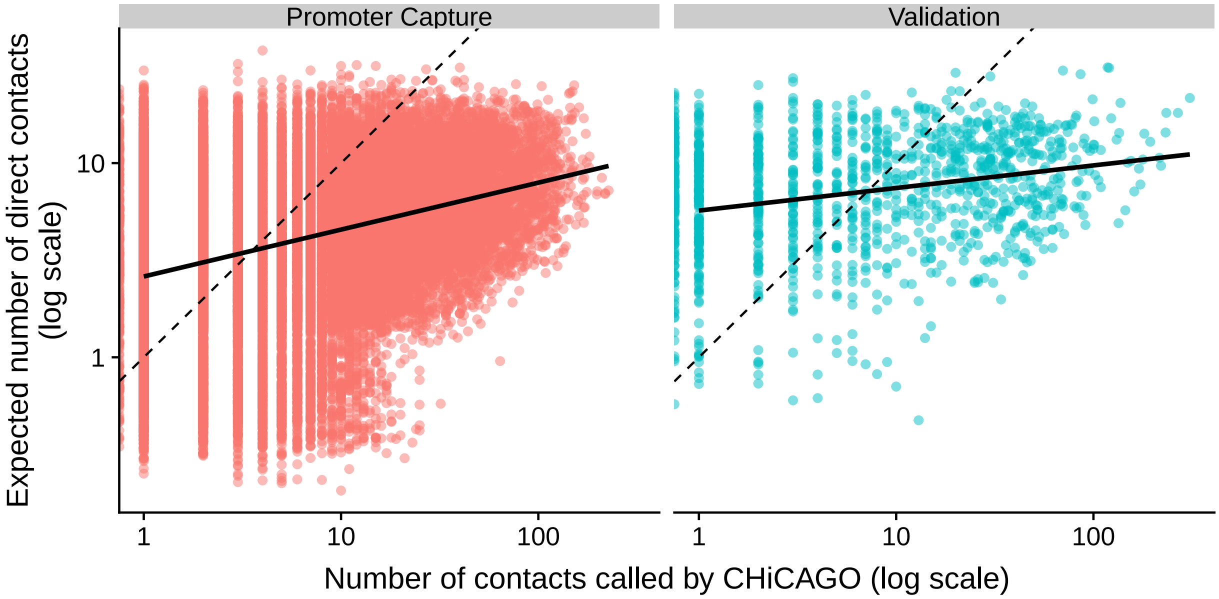


Figure S7: Comparison of number of contacts called by CHiCAGO and the posterior expected number of direct contacts derived from the joint model for each bait that had at least one CHiCAGO significant contact. The dashed line represents $x = y$, the coloured line the best linear fit, and $rho$ gives the Spearman correlation. Results from activated and non-activated cells were very similar and are combined into a single panel.

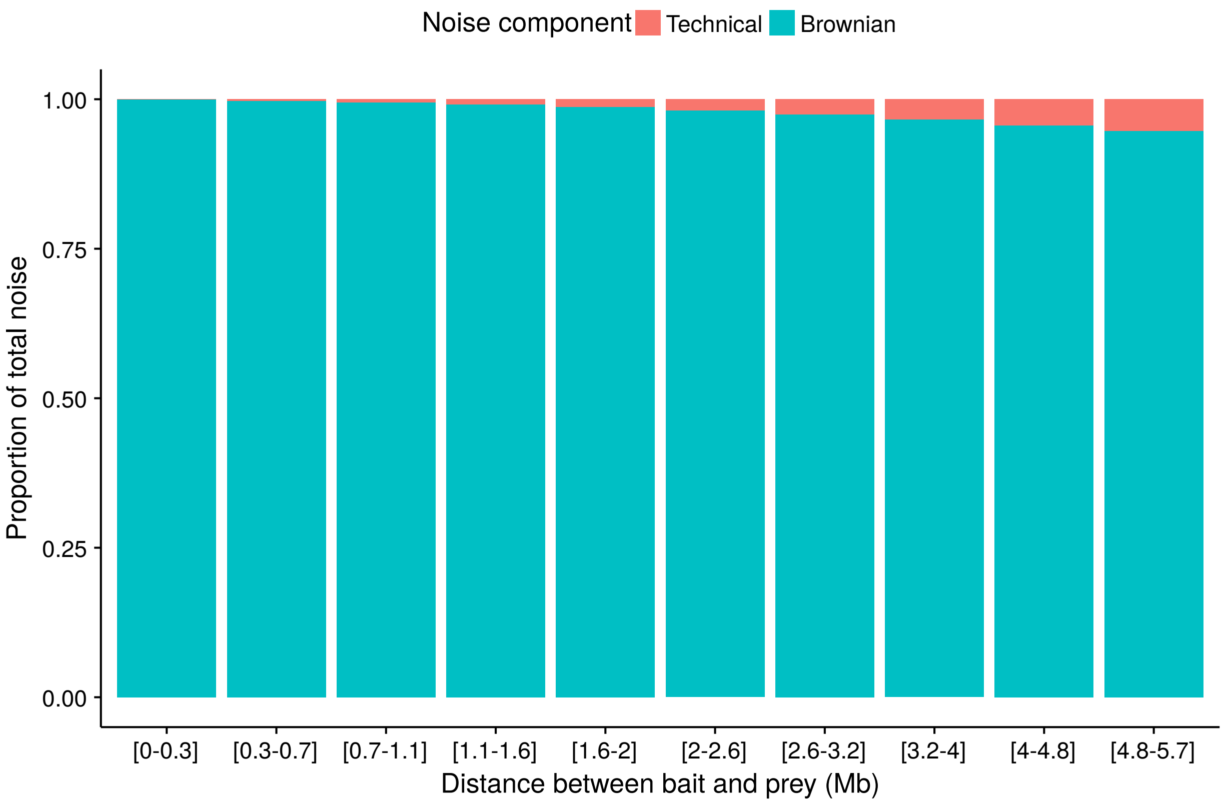


Figure S8: Proportion of residual variance attributable to Brownian or Technical noise in the CHiCAGO analyses, as a function of distance from bait

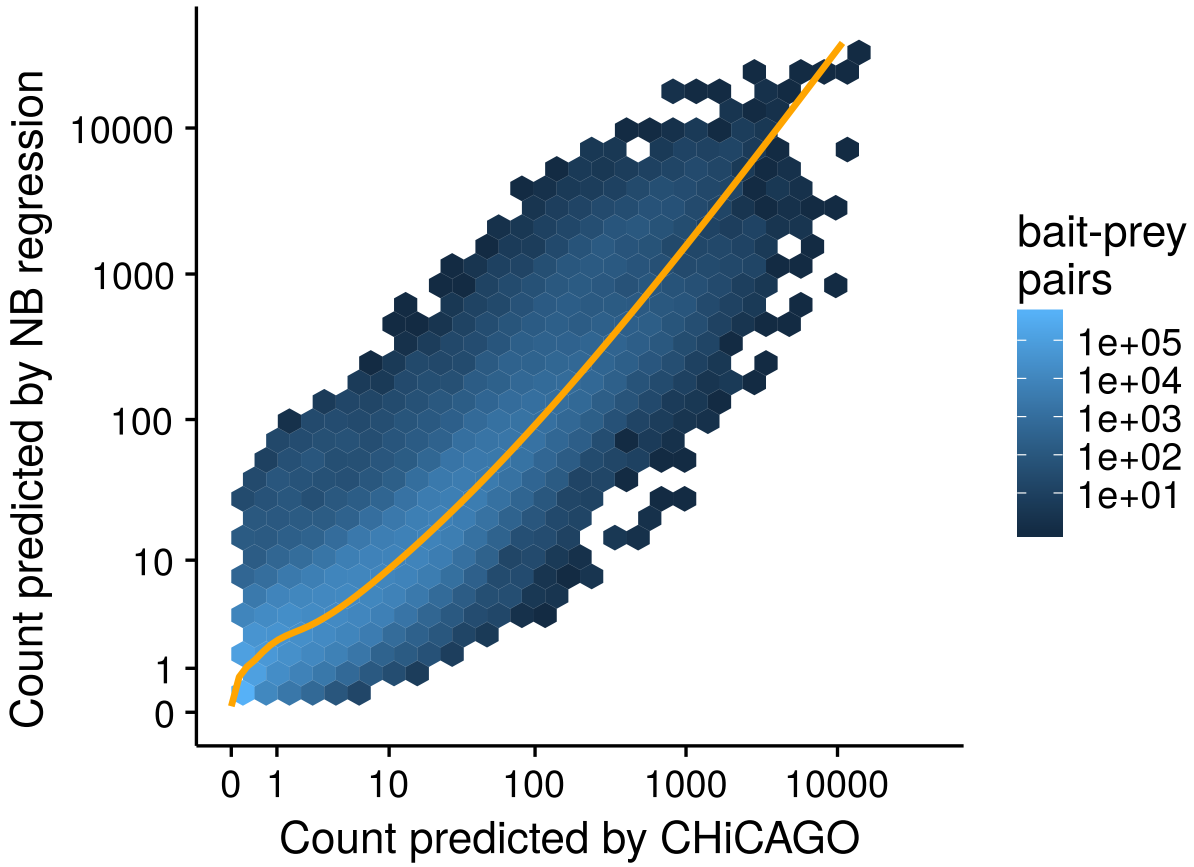


Figure S9: Hexbin plot of predicted counts from regression models used by CHiCAGO and NB regression

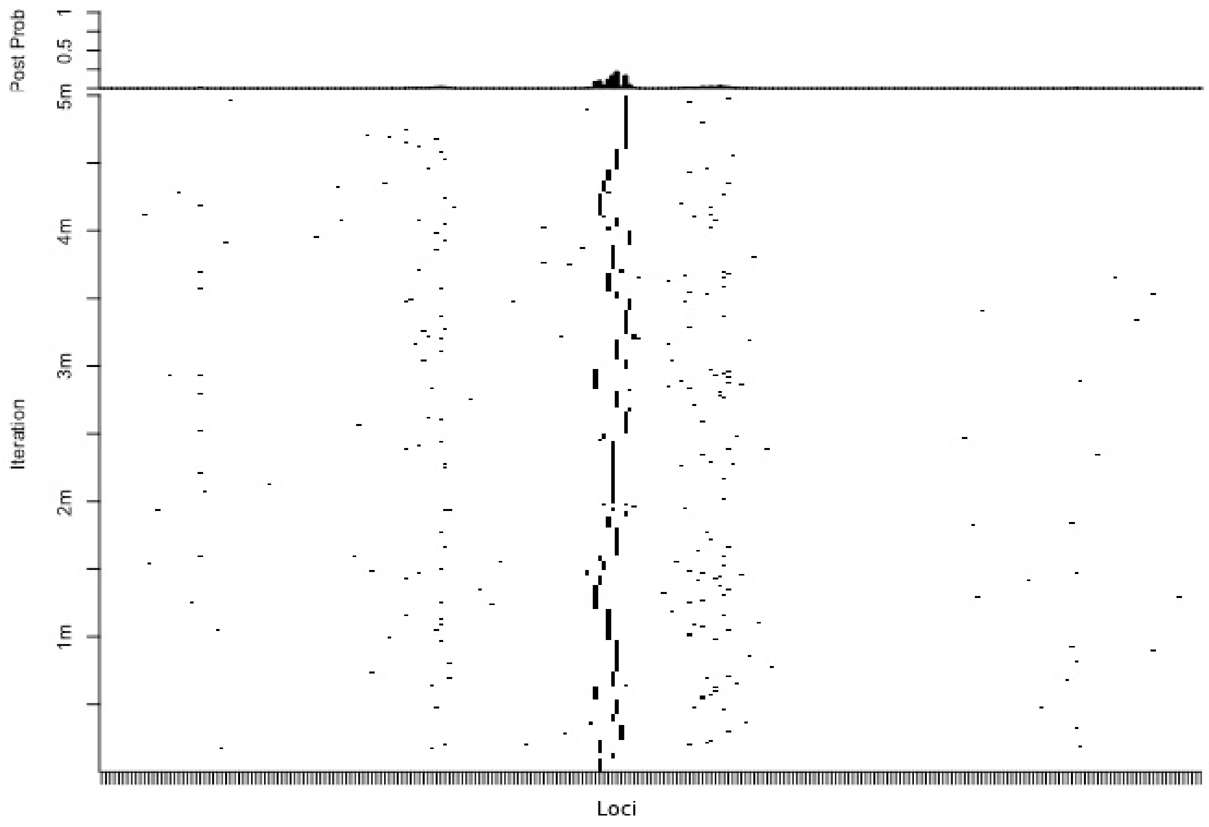


Figure S10: Example autocorrelation plots for one analysis, showing (lower panel) the exploration of the model space as a function of iteration number and (upper panel) summary posterior probabilities of inclusion

Table S1: Summary of distribution of number of preys with CHiCAGO score >5 per bait, amongst those baits with at least one such prey

| Experiment | Min. | 1st Qu. | Median | Mean | 3rd Qu. | Max. |
|---|---|---|---|---|---|---|
| Promoter, non | 40 | 202.0 | 256 | 258.5 | 312.0 | 626 |
| Promoter, act | 33 | 197.0 | 251 | 252.9 | 307.0 | 726 |
| Validation, non | 100 | 268.8 | 338 | 352.3 | 422.2 | 1112 |
| Validation, act | 95 | 268.2 | 346 | 355.9 | 429.2 | 1067 |

Table S2: ΔBIC from the intercept only model for whether the prey fragment overlaps active chromatin states defined by [28] in non-activated and activated cells. The best fitting model (lowest ΔBIC) is highlighted by *. In all cases, a robust clustered model was used to account for repeated observations at the prey fragment.

| **Model** | **Stretch length** | | | |
|---|---|---|---|---|
| | 2-4 | 5-10 | 11-20 | 21+ |
| **non** | | | | |
| MPPC | −364.9 | −278.6 | −112.5 | −98.5 |
| CHiCAGO | −947.0 | −674.2 | −356.6 | −165.2 |
| MPPC + CHiCAGO | * −1111.3 | * −747.4 | * −359.2 | * −175.5 |
| **act** | | | | |
| MPPC | −261.7 | −179.4 | −105.2 | −80.2 |
| CHiCAGO | −1054.6 | −530.2 | * −394.1 | * −194.8 |
| MPPC + CHiCAGO | * −1150.2 | * −568.4 | −393.0 | −194.1 |

Table S3: Distance bins used for NB regression

| bin | Min Dist | Max Dist | Number of fragment pairs |
|---|---|---|---|
| **Promoter capture, non-activated** | | | |
| 1 | 2502 | 607652 | 3579047 |
| 2 | 607653 | 1358092 | 3579041 |
| 3 | 1358093 | 2325619 | 3579043 |
| 4 | 2325620 | 3526852 | 3579046 |
| 5 | 3526853 | 5000000 | 3579038 |
| **Promoter capture, activated** | | | |
| 1 | 2502 | 610510 | 3635118 |
| 2 | 610511 | 1371607 | 3635115 |
| 3 | 1371608 | 2347572 | 3635116 |
| 4 | 2347573 | 3543515 | 3635114 |
| 5 | 3543516 | 5000000 | 3635116 |
| **Validation, non-activated** | | | |
| 1 | 2506 | 649484 | 203449 |
| 2 | 649496 | 1429935 | 203449 |
| 3 | 1429942 | 2371494 | 203449 |
| 4 | 2371495 | 3546440 | 203449 |
| 5 | 3546443 | 4999996 | 203449 |
| **Validation, activated** | | | |
| 1 | 2506 | 626939 | 213282 |
| 2 | 626940 | 1395426 | 213281 |
| 3 | 1395431 | 2331663 | 213282 |
| 4 | 2331665 | 3505263 | 213280 |
| 5 | 3505266 | 4999996 | 213282 |
